# Conductive Organometallic Polymers from Soluble Superatom Ions

**DOI:** 10.1021/acsmaterialslett.5c00925

**Published:** 2025-09-05

**Authors:** Jonathan H. Gillen, My K. Vuong, Daniel W. Paley, Christopher M. Bejger

**Affiliations:** † Department of Chemistry, 14727 The University of North Carolina at Charlotte, 9201 University City Boulevard, Charlotte, North Carolina 28223, United States; ‡ Molecular Biophysics and Integrated Bioimaging Division, 1666 Lawrence Berkeley National Laboratory, Berkeley, California 94720, United States

## Abstract

Superatomic crystals comprising ligand-capped, metal chalcogenide clusters and fullerenes are modular materials that exhibit enhanced electronic, magnetic, and thermal conductivity properties. We find that neutral, M_4_S_4_ (M = Fe, Co) clusters stabilized with *N*-heterocyclic carbenes (NHCs) can transfer charge to C_60_ fullerene to form binary superatomic crystals. Notably, these compounds are soluble in various organic solvents, allowing their properties to be investigated in solution, unlike traditional fullerene-based superatomic crystals. The ion pairs can be further assembled into organometallic polymers using Janus-bis­(NHCs) to cross-link the oxidized M_4_S_4_ units. We show that the superatomic polymers are more conductive than both the precursor superatomic crystals and the polymers containing only neutral M_4_S_4_ clusters. Similar conductivity values can be obtained when neutral M_4_S_4_–NHC polymers are doped with solutions of C_60_ fullerene. These findings demonstrate that next generation superatomic materials can be prepared via the combination of charge transfer and polymerization with appropriate cross-linking agents.

## Graphical abstract

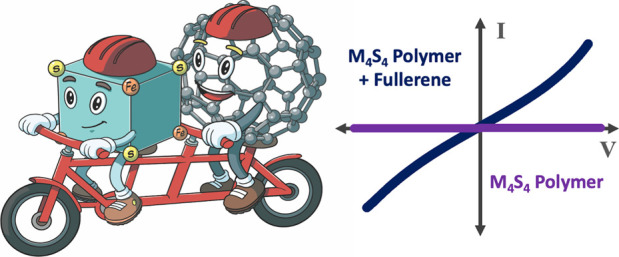

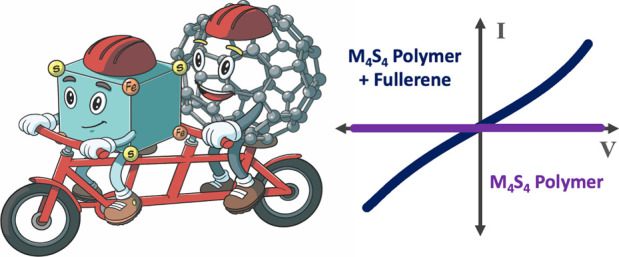

Directing the assembly of superatomic clusters is an important goal in the field of materials chemistry.^1–3^ A fruitful strategy for preparing extended structures of superatoms is assembly through charge transfer.^4–7^ This technique requires electronically complementary cluster building blocks and results in the formation of ionic superlattices in which charged clusters are electrostatically paired.^8^ Many functional, solid-state materials have been prepared using this methodology. In particular, a suite of superatomic crystals containing metal chalcogenide clusters and fullerides are a paragon of this compound class (Figure 1a).^4^ The synthetic process is general and assorted metal–chalcogenide clusters of varying composition and geometry have been employed as electron donors for this purpose, including, for example, Co_6_Se_8_,^4,5^ Cr_6_Te_8_,^4,5^ Ni_9_Te_6_,^4,9^ and Ni_3_Te_2_ ^10^ clusters. Multiple carbon-based nanoclusters, such as C_60_,^4,9,10^ C_70_,^10,11^ and endohedral fullerenes^12^ have also found use as the corresponding acceptors for these superatomic crystals. The resulting hybrid materials exhibit electronic^4,5,10,13^ and thermal transport^14^ as well as magnetic properties^9^ based on the precursor clusters, degree of electron transfer, and packing arrangement. However, superatomic crystals are typically insoluble and isolated as brittle crystals or polycrystalline powders. Thus, further transformation of such materials remains limited and necessitates the use of solid-state chemistry protocols, such as intercalation^13,15^ and exfoliation.^16,17^

**Figure 1:**
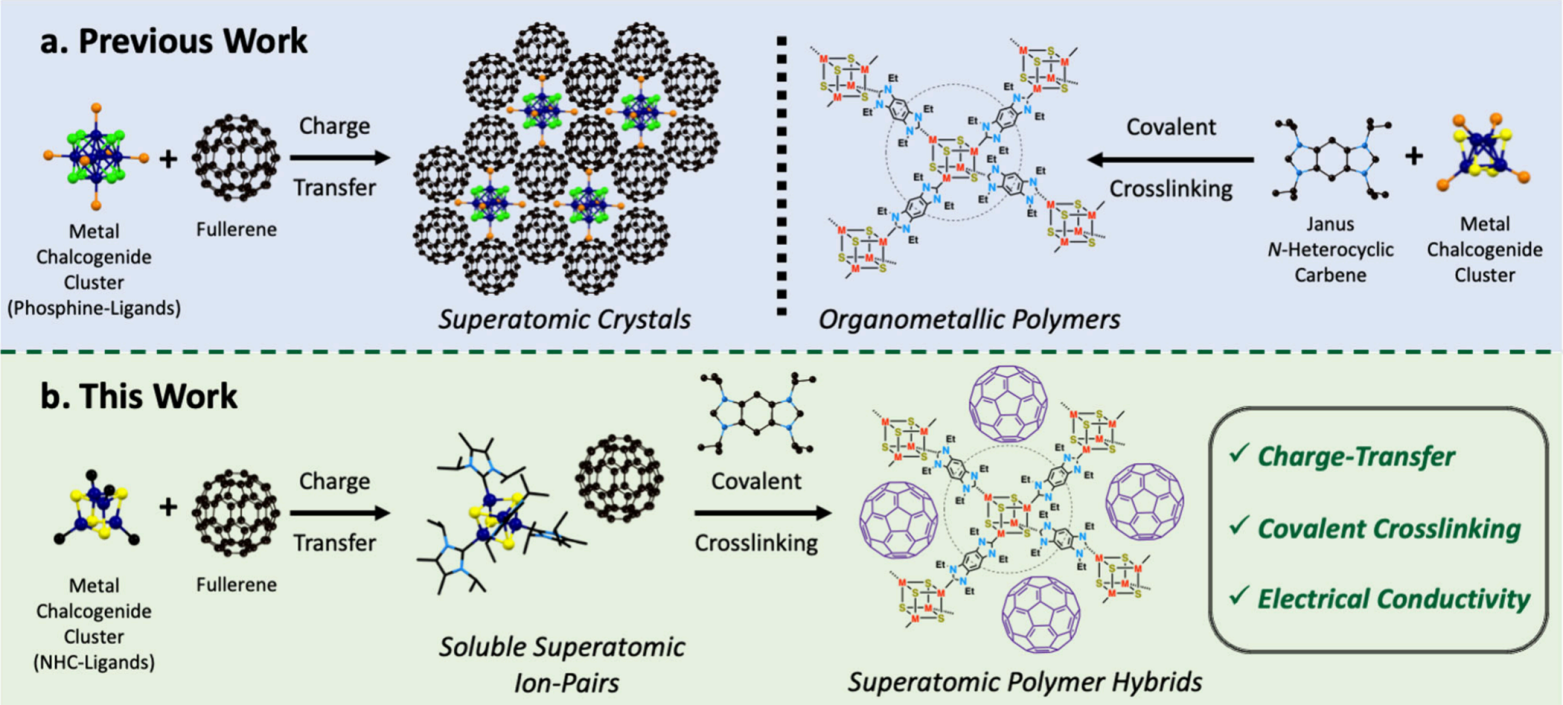
Overview of hybrid approach to superatomic materials. (a) Previous assembly routes to prepare superatomic crystals and organometallic polymers. (b) Stepwise assembly of superatomic polymer hybrids via charge transfer followed by covalent cross-linking.

Superatomic clusters can also be assembled covalently (Figure 1a). Numerous coordination polymers have been prepared using polytopic, bridging ligands to cross-link metal chalcogenide clusters,^18–24^ metal halide clusters,^25–27^ polyoxometalates,^28,29^ and others.^30,31^ These superatomic frameworks represent a significant achievement from a structural perspective. However, superatom-based materials constructed through the combination of cross-linking and charge transfer are rare.^32,33^ This is in contrast to purely organic charge-transfer complexes, which can be hierarchically organized into polymeric materials^34–38^ and supramolecular assemblies.^39^ New design protocols must be realized to unlock the full potential of superatoms as building blocks. Furthermore, a dual covalent-ionic approach may result in superatomic materials with enhanced stability and electronic coupling between cluster units.^40,41^

Here, we show how to prepare multicomponent assemblies of superatoms through synchronized charge transfer and covalent cross-linking (Figure 1b). This is facilitated by the serendipitous realization of soluble, superatomic ion-pair precursors that can be dissolved and further manipulated in solution. Specifically, we find that neutral, [M_4_S_4_]^0^ (M = Fe, Co) clusters with *N*-heterocyclic carbene (NHC) ligands undergo electron transfer with C_60_ fullerene. The cocrystals can be redissolved and polymerized in the presence of Janus biscarbenes that bridge the M_4_S_4_ cores. We also demonstrate that neutral [M_4_S_4_]^0^ polymers undergo charge-transfer when treated with fullerenes. Both pathways yield materials with electrical conductivities greater than the sum of their parts.

Metal–sulfur, cubane-like clusters remain unexplored as precursors for superatomic crystals, despite their natural occurrence^42^ and the extensive investigation of their synthetic counterparts.^43^ The archetypal Fe_4_S_4_ clusters, which are analogues of protein-bound redox centers, are particularly appealing donors for this purpose due to their propensity to electron transfer and ability to be prepared with various ligand types.^44–48^ For instance, certain all-ferrous [Fe_4_S_4_]^0^ cubanes are electron-rich and reversibly ionize at potentials suitable for reducing fullerene.^48^ We initially selected two, isostructural [M_4_S_4_]^0^ clusters (M = Fe (**1**), Co (**2**)) as electron donors, both of which were first reported by Holm and co-workers.^48,49^ These nanoclusters are synthesized in two steps and are isolated with the NHC supporting ligands 1,3-diisopropyl-4,5-dimethylimidazol-2-ylidene (Pr^*i*^_2_NHCMe_2_). It is noteworthy that the metal chalcogenide clusters found in superatomic crystals are traditionally passivated with phosphine ligands.^3^ We found that trialkylphosphine-ligated M_4_S_4_ clusters lack the ionization strength to reduce C_60_. In contrast, NHC-capped Co_4_S_4_ and Fe_4_S_4_ clusters spontaneously transfer electrons to fullerene in solution.

Binary superatomic crystals are grown by mixing solutions of C_60_ fullerene with equimolar mixtures of **1** or **2** in chlorobenzene. After 24 h, long black rods are isolated and washed with toluene (). Crystals of **2·C**_**60**_ are suitable for single-crystal X-ray diffraction (SCXRD) and crystallize in the tetragonal space group *P*4*n*2 (). The structure of **2·C**_**60**_ consists of each cluster in a 1:1 ratio with one accompanying chlorobenzene molecule. The Co_4_S_4_ and C_60_ units are arranged in segregated columns down the *c*-axis (Figure 2a). These columns alternate across both the *a*- and *b*-axes, respectively (Figure 2b). The fullerides form single-bonded (C_60_^–^)_2_ dimers in the solid state, with a C–C bond between neighboring fullerenes down the stacks. Chlorobenzene molecules fill the void space around each cluster and alternate in a head-to-tail manner. There are short contacts (3.397 Å) between the chlorine atoms and C_60_^–1^, which fall in the range of reported C–Cl···π halogen bonding interactions.^50,51^ **2·C**_**60**_ crystals obtained from chlorobenzene cannot be redissolved. We hypothesize that the presence of fulleride dimers and halogen bonding contribute to the robust nature of the structure.

**Figure 2:**
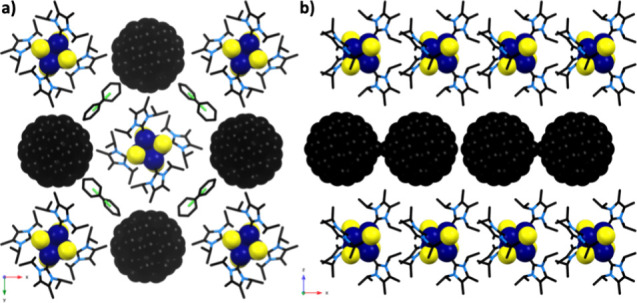
Structure of **2·C**_**60**_ showing (a) the crystal packing looking down the *c*-axis and (b) a single layer view down the *b*-axis. Hydrogen atoms are removed for clarity. Solvent atoms are omitted in panel (b). [Color legend: carbon, black; cobalt, dark-blue; chlorine, green; nitrogen, light-blue; sulfur, yellow.]

The crystal structure of **2**·**C**_**60**_ provides information about the oxidation state of the Co_4_S_4_ cluster. We compared the Co–Co and Co–S bond lengths of **2**·**C**_**60**_ to those of neutral cluster **2** and **2**·BPh_4_ (). Both the Co–Co (2.66 Å) and Co–S (2.22 Å) distances in **2**·**C**_**60**_ are reduced compared to **2** (2.69 and 2.25 Å) and match with the bond lengths in **2**·BPh_4_ (2.66 and 2.22 Å). Thus, the Co_4_S_4_ clusters in **2**·**C**_**60**_ are found to be in the +1 oxidation state.

Charge transfer between the M_4_S_4_ nanoclusters and fullerene also occurs in other solvents. For example, mixtures of **1** or **2** in toluene with C_60_ yield black needles overnight. SEM micrographs of **1**·**C**_**60**_ show rod-like crystals of 50–75 μm lengths (). SCXRD data for **1**·**C**_**60**_ was not obtained, but X-ray diffraction studies confirm that the material is crystalline with the same lattice parameters as **2·C**_**60**_ (). The elemental composition of the co-crystals was also determined using EDS (). The spectra of **1·C**_**60**_ contain C, N, Fe, and S throughout the crystals with the expected equivalent ratio of metal to sulfur.

Remarkably, co-crystals of **1·C**_**60**_ and **2·C**_**60**_ prepared from toluene are soluble in several organic solvents, including, dimethyl sulfoxide, *o*-dichlorobenzene (*o*-DCB), tetrahydrofuran (THF), and 1-methylnaphthalene (Figures 3a and ). Thus, the superatomic ion pairs can be characterized in solution using electrochemical analysis and common spectroscopic methods. The solubility of these materials is unique for fulleride-cluster superatomic crystals. We are aware of only one other related example of a soluble cluster-fulleride superatomic compound: a mixture of [6,6]-phenyl-C_61_-butyric acid methyl ester (PCBM) and Co_6_Te_8_(PPr_3_)_6_.^52^

**Figure 3:**
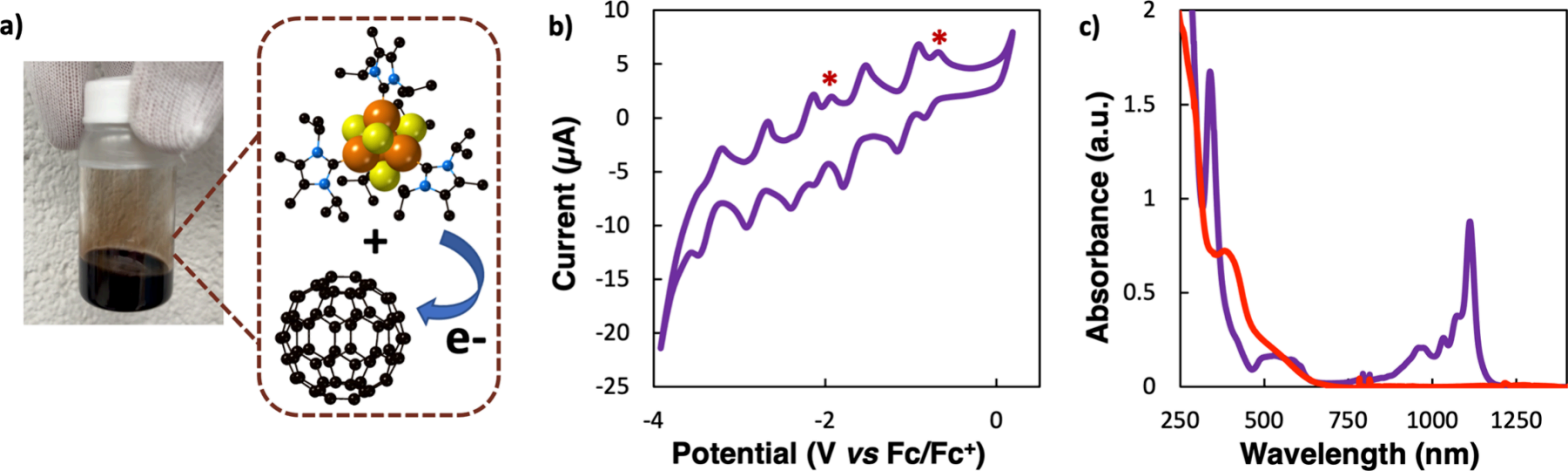
Characterization of **1·C**_**60**_. (a) Photograph of **1·C**_**60**_ in *o*-DCB and graphical depiction of cluster electron transfer. (b) Cyclic voltammogram of **1·C**_**60**_ recorded at 100 mV s^–1^ in THF with TBAPF_6_ using a glassy carbon working electrode. (c) UV–vis–NIR spectra of **1** (red) and **1·C**_**60**_ (purple) in THF.

The electrochemical properties of **1·C**_**60**_ and **2·C**_**60**_ were investigated using cyclic voltammetry (CV) (Figure 3b). Solutions of **1·C**_**60**_ in THF undergo seven reversible redox events across a range of 3 V. Five of these waves correspond to fullerene-based reductions.^53^ We are unable to detect the sixth C_60_ reduction. The two additional waves in the voltammogram of **1·C**_**60**_ at *E*_1/2_ = −0.74 V and −2.0 V vs Fc/Fc^+^ (−0.12 V and −1.38 V vs SHE) are assigned to the Fe_4_S_4_ core.^48^ These values are consistent with the measured oxidations of the neutral Fe_4_S_4_ cluster **1**, which occur at *E*_1/2_ = −0.80 V and −2.0 V vs Fc/Fc^+^ (). The CV of **2·C**_**60**_ in THF also exhibits redox events for both the fullerene and cluster **2** (). The M_4_S_4_ redox events of **2·C**_**60**_ occur at higher potentials (*E*_1/2_ = −0.38 V and −1.63 V vs Fc/Fc^+^ and 0.24 V and −1.0 V vs SHE) than **1·C**_**60**_. This is expected, as the Fe congener **1** is more electron-rich than **2**.^49^ Linear sweep voltammetry measurements were also carried out to obtain the resting potentials of solutions of **1·C**_**60**_ and **2·C**_**60**_ (). The resting potentials were found to be −1.21 V (**1·C**_**60**_) and −1.18 V (**2·C**_**60**_), which verifies that the M_4_S_4_ units are singly oxidized, and the fullerenes are in the −1 oxidation state. Such rich electrochemistry confirms the structural integrity of both the metal–sulfur clusters and C_60_ in these soluble cluster pairs.

The degree of charge transfer between clusters in **1·C**_**60**_ and **2·C**_**60**_ was also obtained spectroscopically. Electronic absorption spectroscopy was first used to probe the oxidation state of the fullerenes. Fullerene redox states are known to have signature absorbance features in the near-infrared (NIR) region that are cation and solvent independent.^54^ The UV–vis-NIR spectrum of **1·C**_**60**_ in THF has a strong NIR transition at ca. 1115 nm with two additional overlapping, higher-energy bands and a fourth feature at 973 nm (Figure 3c). The shape of the absorbances in the NIR region and the values of the two main peaks are consistent with a C_60_^–1^ oxidation state. Specifically, reported spectra of fulleride anions exhibit a dominant peak around 1100 nm and a transition between 930 and 995 nm.^54^ We measured a near-identical UV–vis–NIR spectrum for **2·C**_**60**_ in THF with the fulleride features blue-shifted to 1103 and 953 nm, respectively (). Information regarding the oxidation state of cluster **1** can also be gained by comparing the spectra in Figure 3c. The attenuation of the 375 nm transition in the spectrum of **1** and growth of a new peak at 345 nm in the spectrum of **1·C**_**60**_ is consistent with the change of the [Fe_4_S_4_]^0^ core to the [Fe_4_S_4_]^+^ state.^55^

Nuclear magnetic resonance (NMR) spectroscopy of the dissolved superatomic crystals was performed to further probe the oxidation states of the constituent clusters in solution. The neutral and oxidized forms of parent cluster **2** have distinctive proton-NMR spectra.^49^ Thus, NMR experiments were carried out on solutions of **2·C**_**60**_. The ^1^H NMR spectrum of **2·C**_**60**_ in chlorobenzene-*d*_5_ includes a broad singlet at 10.5 and second peak at 8.58 ppm (Figure 4). These two peaks correspond to methyl protons attached to the imidazolylidene and the methyl protons of the isopropyl groups, respectively. We observe that both peaks are noticeably shifted downfield from the NMR spectrum of the neutral cluster **2**. Furthermore, the chemical shifts of these resonances move toward those observed in the NMR spectrum of the oxidized cluster **2·PF**_**6**_ in CD_3_CN (δ 11.75 and 9.53).^49^ We therefore conclude that the oxidation state of the M_4_S_4_ entity is +1 in **2·C**_**60**_. Analogously, the ^1^H NMR spectrum of **1·C**_**60**_ in chlorobenzene-*d*_5_ also includes two methyl peaks from the NHC ligands that are significantly shifted (>5 ppm), compared to neutral **1** (). These peaks at 3.11 and 6.56 ppm are consistent with the reported values of 3.17 and 6.63 ppm for **1·BPh**_**4**_ in THF.^56^ In accordance, carbon-NMR data corroborates the oxidation state of fullerene in the co-crystals. The ^13^C NMR spectra of **1·C**_**60**_ and **2·C**_**60**_ both include a broad peak centered at 185 and 183 ppm, respectively, which reflects the presence of C_60_^–1^ ().^54^ Notably, the carbon-NMR experiments were run for 24 h, underscoring the stability of the ionic species in solution.

**Figure 4:**
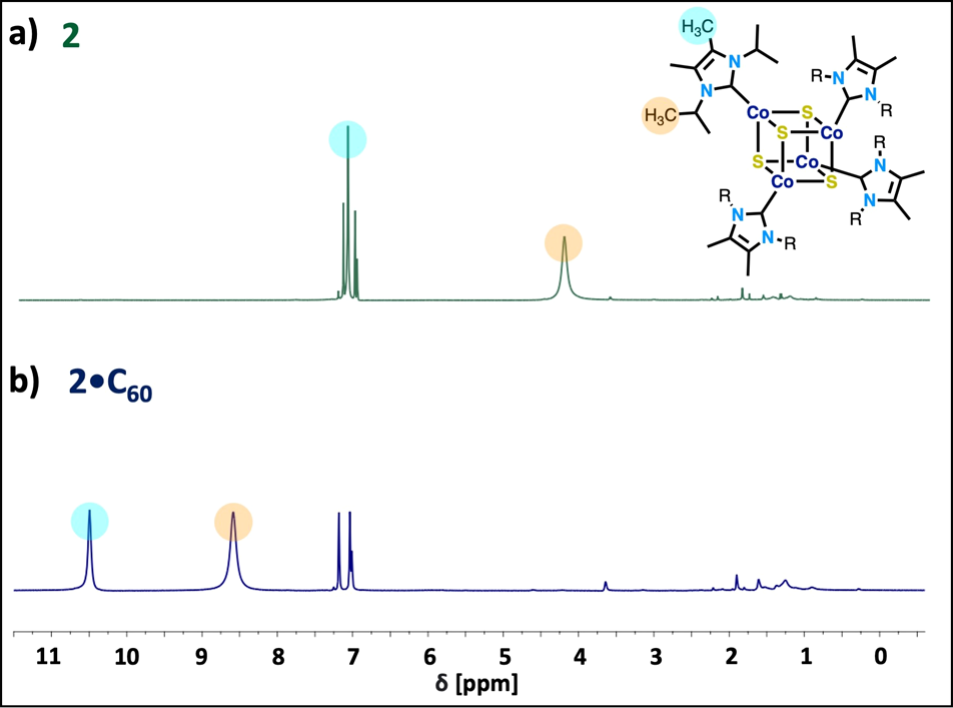
NMR spectroscopy studies of **2·C**_**60**_. (a) ^1^H NMR spectra of **2** and (b) **2·C**_**60**_ in chlorobenzene-*d*_5_. All spectra recorded at 500 MHz.

The inherent solubility of the M_4_S_4_·C_60_ superatomic crystals prompted us to investigate their use as reactants for further synthetic transformations (Scheme 1). Our group recently reported that parent Co_4_S_4_ clusters can be cross-linked with Janus-biscarbenes to generate multidimensional, main-chain organometallic polymers (MCOPs).^18^ Thus, we anticipated that the M_4_S_4_ entities in **1·C**_**60**_ and **2·C**_**60**_ could also be assembled under similar reaction conditions to yield MCOPs with accompanying fullerides. We first investigated the polymerization of **2·C**_**60**_, adapting our synthetical protocol for **MCOP-2**. Solutions of **2·C**_**60**_ in *o*-DCB were mixed with benzo-bis-imidazolylidene (**3**) and heated in a sealed tube at 100 °C (Scheme 1). After 48 h, the solution was clear, and a dark-colored precipitate was isolated by centrifugation inside a nitrogen-filled glovebox. SEM micrographs reveal that **MCOP-2·C**_**60**_ exhibits a similar morphology as **MCOP-2** (). Both materials resemble brittle, amorphous solids lacking distinctive structural regularity. We also observe negligible differences in the measured powder X-ray diffraction (PXRD) patterns of **MCOP-2** and **MCOP-2·C**_**60**_ (). Comparison between the simulated PXRD pattern of **2·C**_**60**_ and the observed pattern of **MCOP-2·C**_**60**_ also indicates that co-crystal impurities are not present in **MCOP-2·C**_**60**_ (). The solid-state UV–vis-NIR absorption spectrum of **MCOP-2·C**_**60**_ includes features of the Co_4_S_4_-MCOP at ca. 500 nm and the fulleride at 970 nm. Additional fulleride peaks above 1000 nm are not observed with certainty due to instrumentation limitations (). The PXRD and electronic absorption data, taken in concert, show that the MCOP can be prepared without measurable structural changes using **2·C**_**60**_ as a precursor.

**Scheme 1:**
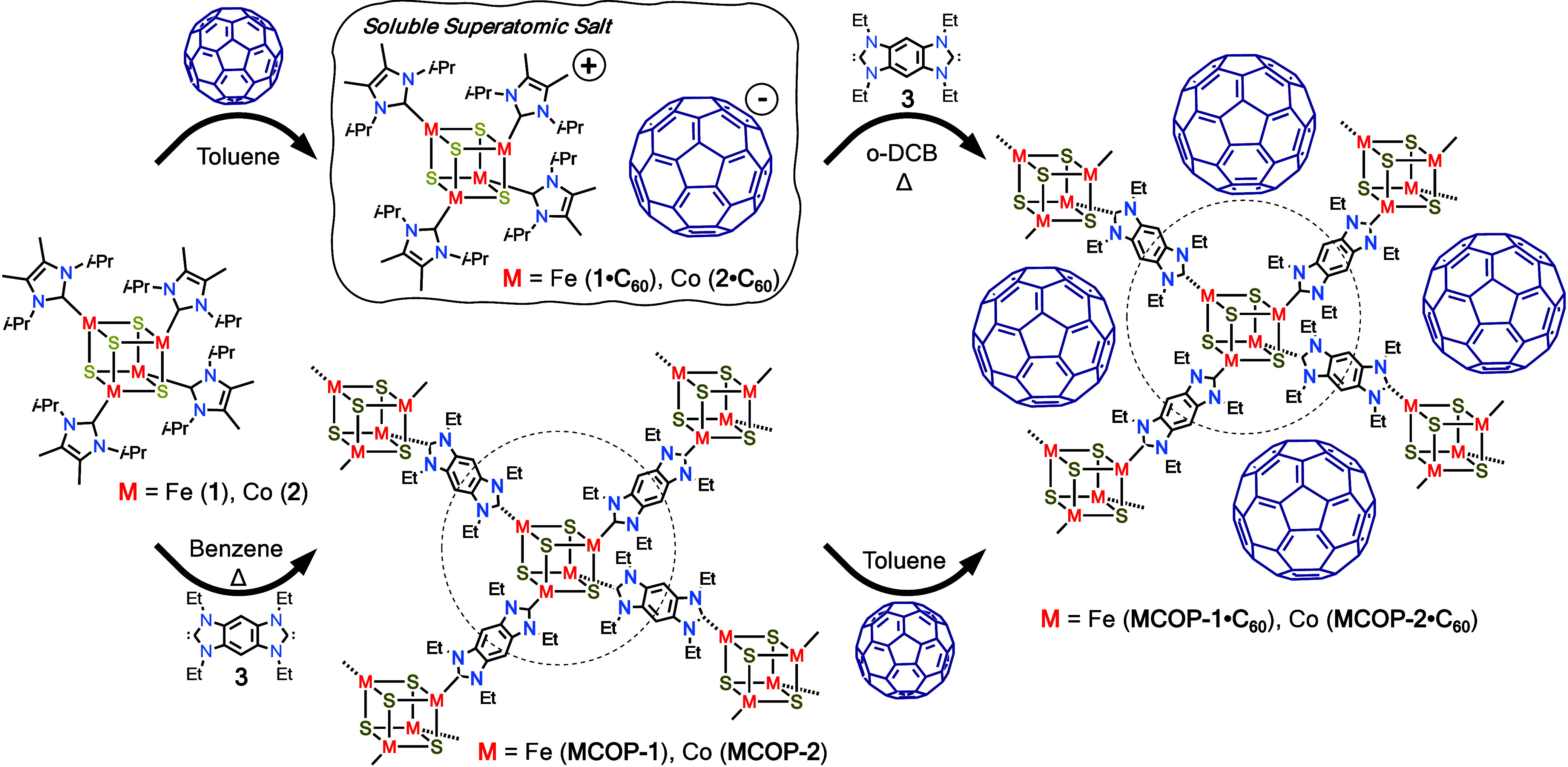
Synthesis of M_4_S_4_·C_60_ Superatomic Crystals, Organometallic Polymers, and Superatomic Organometallic Polymers

The successful copolymerization of **2·C**_**60**_ and **3** motivated us to explore the synthesis of the iron–sulfur analogue. However, the polymer absent-fulleride (**MCOP-1**) was not reported previously. Here, details are provided for its preparation and characterization. (See the .) **MCOP-1** is a deep-blue solid. This polymer was subject to the same characterization methods as **MCOP-2 (** ). PXRD scans of **MCOP-1** contain broad features at low angles like **MCOP-2**, which indicate that it is mostly an amorphous material (). We note that the solid-state UV–vis spectrum of **MCOP-1** is red-shifted compared to **1** (). The shift of the λ_max_ to lower energy is ascribed to the increase in conjugation of the biscarbene-linked polymer compared to the discrete cluster. A similar red shift of the λ_max_ is also observed when **2** is covalently cross-linked by **3**.^18^ Finally, we chemically disassembled the polymer to verify the presence of the Fe_4_S_4_ cluster in **MCOP-1**_._ Depolymerization of **MCOP-1** occurs upon addition of excess monocarbene in a similar fashion to **MCOP-2**.^18^ NMR studies corroborate that the intact Fe_4_S_4_ cluster **1** can be chemically extruded from the solid ().

Formation of **MCOP-1·C**_**60**_ was accomplished using the protocol for **MCOP-2·C**_**60.**_ A black solid forms rapidly when **1·C**_**60**_ is heated in *o*-DCB with **3**. Presence of fulleride in **MCOP-1·C**_**60**_ was again corroborated via absorbance spectroscopy. The spectrum includes peaks at 970 and 1090 nm (). Fullerene incorporation can also be achieved post-synthetically by treating suspensions of **MCOP-1** or **MCOP-2** in toluene with C_60_ fullerene (). Charge transfer can be monitored visually with the naked eye. Purple-colored solutions of fullerene in toluene are clear after 24 h of exposure to the solid MCOPs ().

We measured the electrical conductivity on pressed pellets of the individual clusters, superatomic crystals, and MCOPs with and without fulleride. Measurements were carried out using a two-point probe apparatus under nitrogen.^57^ We observe that MCOP materials are more conductive than the precursor clusters by several orders of magnitude (Figure 5 and Table 1). A similar trend was also shown by Brozek and co-workers, who recently reported large increases in conductivity through the linear polymerization of Fe_4_S_4_^2+^ clusters and bis­(NHCs).^58^ In addition, cocrystals **1·C**_**60**_ and **2**·**C**_**60**_ are considerably more conductive than the precursor clusters. Comparable gains in conductivity have been observed in related superatomic solids comprising fullerides.^4,7,10^ The incorporation of fulleride results in further conductivity enhancements for both the cobalt and iron MCOPs. Specifically, the polymers prepared from **1·C**_**60**_ and **2·C**_**60**_ are 40 and 1500 times more conductive, respectively, than the parent MCOPs (Table 1). MCOPs soaked in solutions containing an excess of fullerene (**MCOP-1**_**doped**_ and **MCOP-2**_**doped**_) also exhibit conductivities approaching that of the polymers prepared from superatomic ion pairs (Figure 5 and ). We surmise that fulleride is more thoroughly integrated into the polymers when the binary cocrystals are used as precursors. PXRD patterns of **MCOP-2** also remain unchanged after doping with fullerene (). This indicates that gains in conductivity are not based on structural changes of the polymer but rather electronic contributions of charge-transfer and presence of fullerene.

**Figure 5:**
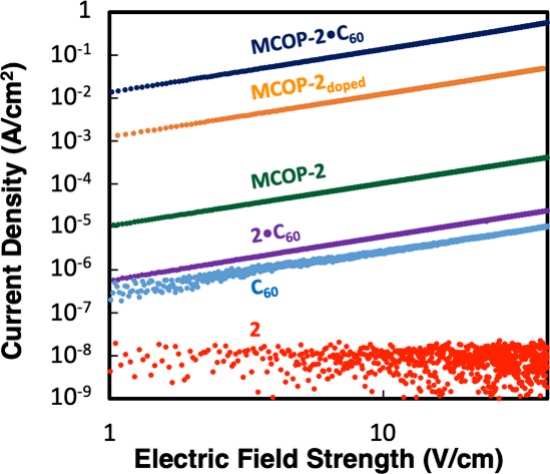
Electrical properties of pressed pellets. Plots of current density versus electric field strength (*J*–*E*) curves at 297 K.

**Table 1:** Electrical Conductivities of Clusters, Cocrystals, and MCOPs in (mS/cm)

	Electrical Conductivity (mS/cm)	
material	**1**	**2**
M_4_S_4_(NHC)_4_ ^a^	6.01 × 10^–7^	1.94 × 10^–7^
M_4_S_4_(NHC)_4_·**C_60_**	2.28 × 10^–4^	5.93 × 10^–4^
MCOP	3.10 × 10^–5^	1.04 × 10^–2^
**MCOP_doped_**	7.47 × 10^–4^	1.24
**MCOP·C_60_**	1.24 × 10^–3^	16.1

a The high electrical resistivities of **1** and **2** inhibited the accurate determination of conductivities with certainty.

Both cobalt and iron materials in this series exhibit similar trends in conductivity. However, the cobalt-based MCOPs are significantly more conductive than the iron congeners. We attribute this to differences in the polymer structure related to the metal–sulfur fragments. Specifically, all-ferrous Fe_4_S_4_ clusters are notoriously challenging to isolate and have a propensity toward core aggregation upon ligand dissociation. The self-assembly of [Fe_4_S_4_]^0^ cores has been observed with various ligand types, including phosphines,^59^ NHCs,^48^ and thiolates.^60^ This results in the formation of stable, edge-fused clusters such as Fe_8_S_8,_ Fe_12_S_12_, and Fe_16_S_16_.^45,48,59,60^ In contrast, [Co_4_S_4_]^0^ cores are less prone to aggregate. For example, the phosphine-supported [Co_4_S_4_]^0^ cluster can be isolated and handled as a crystalline solid,^49^ while the iron analogue is only observed transiently in solution.^45,59^ In addition, the [Fe_8_S_8_]^0^ dicubane with NHC ligands has been prepared whereas the Co_8_S_8_ equivalent is unknown.^48^ Formation of these polycubane “defects” during polymer synthesis could potentially disrupt the electronic and physical structure of the material and increase overall resistivity of the iron-based MCOPs.

Finally, we prepared **MCOP-2·BF**_**4**_ to decouple the oxidation state of the cluster from the impact of fullerene on the electrical properties of the polymer. Pressed pellets of **MCOP-2·BF**_**4**_ are approximately 15 times more resistive (7 × 10^–4^ mS/cm) than **MCOP-2** (). Thus, we conclude that oxidation of the cluster does not independently improve conductivity in the MCOPs and that the presence of fulleride is critical for effective charge transport in these materials. Charge transfer with fullerene alone, however, is insufficient for achieving high conductivities. This is corroborated by the mediocre performance of cocrystals **1·C**_**60**_ and **2·C**_**60**_, which contain fulleride but are not cross-linked. Superior electrical conductivities in both cases arise from the synergistic effects of cross-linking and fulleride doping.

In summary, all-ferrous Fe_4_S_4_ clusters and their cobalt-analogues were found to spontaneously reduce C_60_ to yield solution-processable superatomic ions. The unprecedented solubility of these cluster ion pairs allows us to characterize them in both the solution and the solid state. Furthermore, the superatomic solutes can be covalently polymerized when treated with rigid, bis­(NHC) ligands. The resulting ternary materials comprise cluster units that are covalently and ionically coupled. This gives rise to electrical conductivity properties that far surpass those of the discrete building blocks and of the materials assembled from only through-bond or charge-transfer interactions. We envision that this dual-assembly approach may be applied to other superatomic materials to enhance electronic coupling between clusters. The investigation of different bridging linkers, as well as various metal-chalcogenide and fullerene precursors, are currently being studied in our laboratory.

## Supplementary Material





## Notes

Crystal structure for **2**·**C**_**60**_ (CIF)
Synthesis, crystal data, photographs, SEM micrographs, PXRD, UV–vis-NIR, NMR, and *I*/*V* data (PDF)

## ABBREVIATIONS

NHC: *N*-heterocyclic carbene
MCOP: main-chain organometallic polymer
SCXRD: single-crystal X-ray diffraction
Fc: ferrocene
PXRD: powder X-ray diffraction
SHE: standard hydrogen electrode
*o*-DCB: *o*-dichlorobenzene
THF: tetrahydrofuran
