# Supplementary material for: Conductive Organometallic Polymers from Soluble Superatom Ions
Source: ACS Mater Lett. 2025 Sep 5;7(10):3363–9. doi: 10.1021/acsmaterialslett.5c00925 (PMC12505377; doi:10.1021/acsmaterialslett.5c00925)
Supplement: Supplementary file 2 [file tz5c00925_si_002.pdf]

# Conductive Organometallic Polymers from Soluble Superatom Ions

*Jonathan H. Gillen,*[†] *My K. Vuong,*[†] *Daniel W. Paley,*[‡] *and Christopher M. Bejger**[†]

†Department of Chemistry, The University of North Carolina at Charlotte, Charlotte, North Carolina 28223, United States

‡ Molecular Biophysics and Integrated Bioimaging Division, Lawrence Berkeley National Laboratory, Berkeley, California 94720, United States

**Supporting Information**

I. Chemicals, Synthetic Details, & General Information ............ S3
II. Scanning Electron Micrographs of **1•$C_{60}$** and **2•$C_{60}$** ............ S7
III. X-ray Diffraction for toluene crystallized **1•$C_{60}$** and **2•$C_{60}$** ............ S7
IV. Energy dispersive X-ray spectrograph of **1•$C_{60}$** ............ S8
V. Photographs of Dissolved Superatomic Crystals **1•$C_{60}$** ............ S9
VI. Electrochemical analysis of **1•$C_{60}$** and **2•$C_{60}$** ............ S9
VII. UV-Vis-NIR Absorption Spectroscopy of **2•$C_{60}$** ............ S11
VIII. NMR Spectroscopy of Dissolved Superatomic Crystals **1•$C_{60}$** and **2•$C_{60}$** ............ S11
IX. Characterization of **MCOP-2•$C_{60}$** ............ S13
X. Characterization of **MCOP-1** ............ S14
XI. UV-Vis-NIR studies of MCOPs Postsynthetically Doped with Fullerene. ............ S17
XII. Images of MCOPs Postsynthetically Doped with Fullerene ............ S18
XIII. Electrical Conductivity Measurements ............ S18
XIV. X-ray Diffraction Procedures and Tables ............ S20
XV. References ............ S22

## I. Chemicals, Synthetic Details, & General Information

All manipulations were performed under an inert atmosphere of dry $N_2$ using a Schlenk line or MBraun UniLab Pro glovebox unless otherwise noted. Tetrahydrofuran (THF), toluene, dichloromethane (DCM), hexane, and diethyl ether used were dried and purged with $N_2$ on an MB-SPS solvent purification system from MBraun. Chlorobenzene anhydrous (99.8%, Aldrich), *o*-dichlorobenzene anhydrous (Drisolve), and acetonitrile (Drisolve) were freeze pump thawed 3-4x. NaH 60% in mineral oil (Aldrich) for imidazolium deprotonation was washed thoroughly over a medium filter with hexane under a $N_2$ atmosphere and pumped dry. All other chemicals were purchased from commercial sources and used as received, including N,N-dimethylformamide anhydrous/under argon (99.8%, Alfa Aesar), 1,2,4,5 benzene tetrahydrochloride (95%, Aldrich), formic acid (96%, Alfa Aesar), hydrochloric acid (Macron Fine Chemicals), bromoethane (98% Alfa Aesar), sodium carbonate monohydrate (99.5%, Alfa Aesar), anhydrous potassium carbonate (99%, EMD), benzimidazole (98%, Acros Organics), cobalt (II) chloride anhydrous (99.7%, Alfa Aesar), iron (II) chloride anhydrous (99.5%, Alfa Aesar), triisopropylphosphine (98%, Alfa Aesar), bis(trimethylsilyl)-sulfide (95%, Acros Organics), KO*t*Bu (potassium tert-butoxide, 98%, Aldrich), and potassium chunks in mineral oil (98%, Alfa Aesar).

***$Fe_4S_4(Pr^i{}_2NHCMe_2)_4$*** **(1)**. Dichlorobis(triisopropylphosphine)iron(II) [$FeCl_2(PPr^i{}_3)_2$] was first prepared following a modified procedure and used as a crude solid without characterization.[1] Specifically, the mixture of $FeCl_2$ (278 mg, 2.20 mmol), triisopropylphosphine (2.16g, 13.2 mmol) , and benzene (12.5 mL) were stirred for three hours at 60 ºC in a sealed tube. The tube was then transferred to an oven and heated to 100 ºC for 48 hours. The solution was then filtered hot inside a glovebox and dried in vacuo to yield a light-lavender colored solid (945 mg). Then, $Fe_4S_4(Pr^i{}_2NHCMe_2)_4$ cluster was prepared following a modified procedure by Holm and coworkers.[2] A solution of [$FeCl_2(PPr^i{}_3)_2$] (0.945 g, 2.11 mmol) in THF (21 mL) was added into a solution of $(Me_3Si)_2S$ (0.754 g, 4.22 mmol) in THF (21 mL). The reaction was stirred for 2 days. The resulting solution was pumped dry to give a brown oily residue, which was dissolved in 21 mL benzene and filtered. The solution was then treated with a solution of $Pr^i{}_2NHCMe_2$ (0.760 g, 4.64 mmol) in benzene (6 mL). The brown mixture was stirred for 4 days and subsequently filtered. The filtrate was vapor diffused with hexane for 3 weeks. Yield: 0.30 g, 70%. $^1H$ NMR ($C_6D_6$): δ 19.43 (1), 12.45 (br, 2). Methine proton signals were not observed.

***$Co_4S_4(Pr^i{}_2NHCMe_2)_4$ (2).*** This cluster was synthesized according to Holm and coworkers from the $Co_4S_4(P^iPr_3)_4$.[3] In the glove box, $CoCl_2$ (260 mg, 2.00 mmol) was stirred with a solution of triisopropylphosphine (654 mg, 4.00 mmol) in THF (10 ml) for one hour followed by dropwise addition of a solution of bis(trimethylsilyl)-sulfide (0.451 mg, 2.40 mmol) in THF (10 mL). The resulting dark solution was stirred for 2 days and dried under vacuo. The dark oily residue was dissolved in THF (5.5 mL), syringe filtered and layered with MeCN (24 mL) for 7 days. The resulting black crystals were washed with MeCN and hexane and dried under vacuo yielding $Co_4S_4(P^iPr_3)_4$ (243 mg, 0.242 mmol). The cluster is isolated as a dark-black crystalline solid and the purity is verified using $^1H$-NMR spectroscopy.

***Janus bis(NHC)* (3) .** The free Janus biscarbene was prepared following a modified procedure by Bielawski and coworkers.[4] The solid imidazolium dibromide (130 mg, 0.30 mmol), NaH (28.7 mg, 1.20 mmol), and KOtBu (2.5 mg, 22 μmol) were suspended in THF (5 mL) and stirred for 20 h. The resulting suspension was syringe filtered and dried in vacuo to get dark red solid product. Yields vary between 20-50%.

***1•C₆₀.*** Cluster **1** (16.7 mg, 0.0155 mmol) was first dissolved in toluene (5 mL). In a separate vial, a purple solution of $C_{60}$ (11.2 mg, 0.0155 mmol) was prepared by stirring in toluene (5 mL). Then, the purple $C_{60}$ solution was syringe filtered and added into a 40-dram vial. A 2.5 mL toluene intermediate layer was added on top of $C_{60}$ solution. Next, the solution of **1** in toluene was syringe-filtered and slowly added as a top layer. The vial was left undisturbed for two days. As a result, long needle-crystals were formed at the bottom of the vial (71.4 %). These crystals were washed with toluene and dried in vacuo. $^1$H NMR (500 MHz, chlorobenzene-$d_5$): δ 20.11 (s, 6H), 12.24 (s, 12H). Methine peak not observed. $^{13}$C NMR (125 MHz, chlorobenzene-$d_5$): δ 185.24 (s, $C_{60}^{-1}$), 153.25, 79.37, 42.31, 9.46, 7.57, 6.53.

***2•C₆₀.*** Cluster **2** (26 mg, 0.0240 mmol) was first dissolved in toluene (5 mL) and syringe filtered. In a separate vial, a purple solution of $C_{60}$ (30 mg, 0.0416 mmol) was prepared by stirring in toluene (10 mL) overnight. Then, the purple $C_{60}$ solution was syringe filtered and added into a 4-dram vial. Next, the solution of **2** in toluene was slowly added as a top layer. The vial was left undisturbed for two days. Long needle-crystals were formed at the bottom of the vial. These crystals were washed with toluene and dried in vacuo (yield: ≈50 mg, 96%. Theoretical s without toluene in unit cell is 43 mg and with toluene is 52mg). Single crystals can be prepared by dissolving both clusters in chlorobenzene in high concentration and allowing the solution to sit for 48 hours. $^1$H NMR (500 MHz, chlorobenzene-$d_5$): δ 10.45 (s, 6H), 8.54 (s, 12H), -4.15 (br s, 2H). $^{13}$C NMR (125 MHz, *o*-dichlorobenzene-$d_4$): δ 184.57 (s, $C_{60}^{-1}$), 127.55, 66.14, 32.11, 23.23, 15.96, 14.68, 13.24, 10.59.

***MCOP-1.*** Cluster **1** (59 mg, 0.0547 mmol) and Janus bis(NHC) **3** (37 mg, 0.137 mmol) were weighed into two, separate 20 mL vials, and each was dissolved in benzene (7 mL). The solutions were transferred and mixed into a Chemglass pressure vessel and sealed under a $N_2$ atmosphere. The vessel was placed in an oven at 100 °C. After 2 days, the resulting dark blue solid was separated by centrifugation and washed with THF inside the glovebox. Pumping dry under vacuum produced solid with substantially less volume. The reaction can also be carried out in toluene or dichlorobenzene.

***Depolymerization of MCOP-1.*** A suspension of polymer in deuterated benzene (1.2 mL) was mixed with a solution of excess free carbene $Pr^i_2NHCMe_2$ (~20 equiv., 1.2 mL) in a 2-dram vial. The solution was stirred for two days inside the glovebox on a hotplate at 50 ºC. During this time, the majority of the solid dissolved and the solution turned dark, reddish brown. The solution was allowed to sit for several hours before an aliquot (1 mL) was taken from the top of the reaction mixture and analyzed by NMR. The $^1$H NMR spectrum of the depolymerized polymer exhibits the peaks associated with discrete cluster **(1)** at δ 19.43 (1), 12.45 (br, 2) in $C_6D_6$.

***MCOP-2.*** The polymer can be prepared from the phosphine- or NHC-ligated cluster ($Co_4S_4(P^iPr_3)_4$ or **2**). See previous report for full synthetic details.[5]

**MCOP-1•$C_{60}$** and **MCOP-2•$C_{60}$**. Both polymers were prepared in the same fashion. A synthetic prep for **MCOP-1•$C_{60}$** is as follows: Cocrystal **1•$C_{60}$** (29 mg, 0.016 mmol) was dissolved in dichlorobenzene (7 mL) and syringe filtered into a solution of **3** (10 mg, 0.035 mmol) in dichlorobenzene (7 mL). The mixture was heated to 100 ºC for 3 days. The solution turns clear after this time and a dark precipitate is isolated via centrifugation. The solid is washed repeatedly with dichlorobenzene and toluene and then dried in vacuo.

***Post Synthetic MCOP Doping.*** A saturated solution of $C_{60}$ in toluene is prepared and syringe filtered. This solution is added to **MCOP-1** or **MCOP-2** and allowed to sit overnight. This reaction is performed on fresh MCOP (≈20 mg) and a saturated solution of $C_{60}$ in toluene (≈3 mL). The resulting suspension is centrifuged, washed with toluene, and dried in vacuo to yield dark powders (**MCOP-1$_{doped}$** or **MCOP-2$_{doped}$**).

***MCOP-2•$BF_4$.*** The oxidized cluster was first prepared according to a literature prep from $Co_4S_4(P^iPr_3)_4$ (153 mg, 0.152 mmol) and $[Cp_2Fe](BF_4)$.[3] (0.044 mg, 0.163 mmol). The MCOP was synthesized in the same manner as **MCOP-1•$C_{60}$** and **MCOP-2•$C_{60}$**: **2•$BF_4$** (45 mg, 0.038 mmol) was dissolved in 5 mL *o*-dichlorobenzene, syringe filtered and added to 2.5 equivalents of **3** (26 mg, 0.095 mmol) dissolved in 5 mL *o*-dichlorobenzene. The mixture was placed inside of a Chemglass pressure vessel and heated to 100 ºC overnight. The dark precipitate was collected by centrifugation and washed with dichlorobenzene and toluene and then dried overnight under reduced pressure.

***Characterization***

*Nuclear Magnetic Resonance (NMR) Spectroscopy*
NMR spectra were obtained on a Jeol ECA-500 NMR for $^1H$ (500 MHz) and $^{13}C$ (125 MHz). NMR tubes were prepared using deuterated solvents purchased from Cambridge Isotope Laboratories >95% and sealed by parafilm or using a J-Young needle valve.

*Electrochemistry*
All electrochemical measurements were obtained on a Gamry 1000E potentiostat and were recorded on Gamry Framework Software (Version 7.02). Analysis of the data was done using Gamry Echem Analyst (Version 7.02). Cyclic voltammetry was performed with a three-electrode setup with a working glassy carbon electrode (3.0 mm), a platinum counter electrode, and a platinum wire as a pseudo-reference electrode.

*Electrical Conductivity*
Conductivity measurements were taken under inert atmosphere using a homemade device comprised of a thick-walled glass capillary (3 mm inner diameter, 9 mm outer diameter, 36 mm length) with two metal rods (3 mm diameter) compressed with a small trigger clamp.[6] Pellet width measurements were taken with a Mitutoyo 293342-30 Digimatic Outside Micrometer (50.8-76.2 mm) with 0.001 mm resolution. Pellet thicknesses are as follows: **1**, 0.576 mm; **1•$C_{60}$**, 0.706 mm,

*Electronic Absorption Spectroscopy*
A Cary 300 Bio UV-Visible Spectrometer running Scan v1.00(6) was used to collect the UV-Vis spectra with solutions of THF inside of sealed quartz cuvettes to maintain an air free atmosphere. The dried MCOPs with and without $C_{60}$ were run as powdered solids, sealed between two slides, and suspended in bSi-201 quick cure epoxy to prevent oxidation. Backgrounds were taken on a reference sample of epoxy between two slides.

*Scanning Electron Microscopy and Energy Dispersive X-ray Spectroscopy*
Scanning electron micrographs were obtained on a JEOL JSM-6460LV SEM instrument using a 5-15kV accelerating voltage while collecting data through the SEM Control User Interface (Version 6.21). Energy dispersive X-ray spectrographs were taken using the built in EDAX and worked up using the APEX software. SEM samples were pumped down and placed on a copper or carbon tape background with a 60 second air exposure while being loaded. During this exposure, samples of **MCOP-1** and **MCOP-2** would occasionally produce smoke from an exothermic reaction. **MCOP-1•$C_{60}$** and **MCOP-2•$C_{60}$** did not undergo rapid reactions in air to produce smoke.

*Single Crystal X-ray Diffraction*
Single Crystal X-ray diffraction was performed using an Oxford Diffraction Gemini A Ultra SCXRD. Samples were dried for 30 min by vacuum and suspended in Nujol mineral oil before being transferred to the sampling stage and mounted on loops in a stream of $N_2$.

*Powder X-ray Diffraction*
Powder X-ray diffraction patterns were obtained from 11-BM and 17-BM APS Synchrotron Beamline (Bending Magnet Source) at the Argonne National Laboratory (Lemont, Il, USA). X-ray samples were pumped dry, ground by mortar and pestle, and packed into a 1.105 mm outer diameter, 0.1 mm wall Kapton capillary inside a nitrogen glovebox. The capillary ends were then sealed with bSi-201 quick cure epoxy and allowed to cure for at least 24 hours. High resolution synchrotron powder diffraction data were collected at 295 K using beamline 11- BM at the Advanced Photon Source (APS), Argonne National Laboratory using an average wavelength of $\lambda = 0.4581$ Å. Discrete detectors covering an angular range from 0.5 to 50° 2θ, with data points collected every 0.001° 2θ and scan speed of 0.01°/s. The powders were rotated during the measurement at ~50 Hz (11- BM). Synchrotron powder diffraction data from 17-BM was run using an average wavelength of $\lambda = 0.6086$ Å.

## II. SEM Micrographs of 1•$C_{60}$ and 2•$C_{60}$

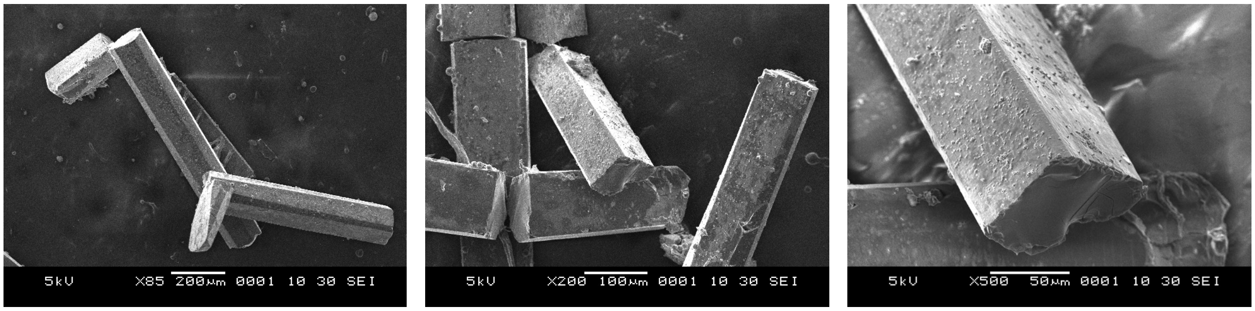


**Figure S1**. SEM micrographs of single crystals of **2•$C_{60}$** grown from chlorobenzene.

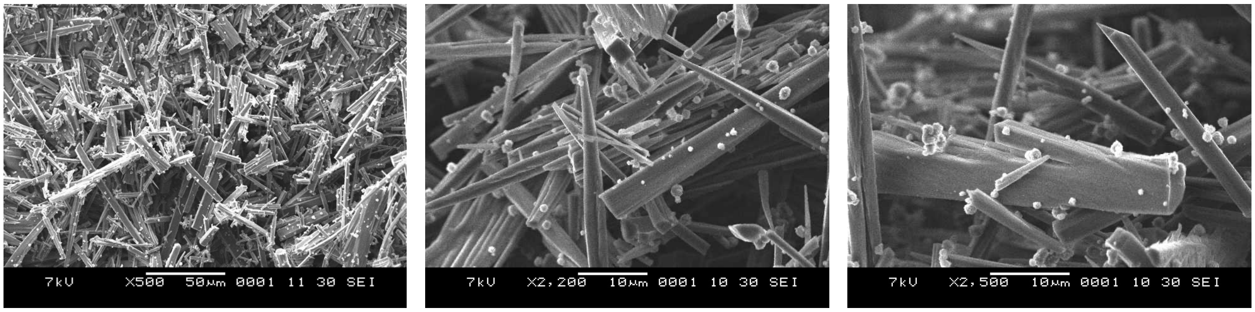


**Figure S2**. SEM micrographs of **1•$C_{60}$** grown from layering solutions of toluene.

## III. X-ray Diffraction

**a.**

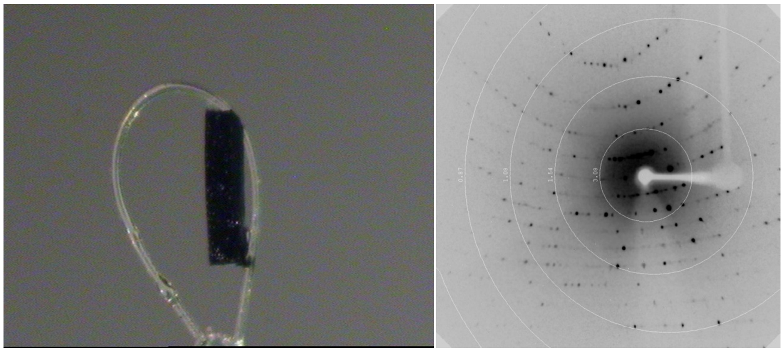

Crystal data and structure refinement for Fe4S4 C60_auto.

| | |
|---|---|
| Identification code | **1•$C_{60}$** |
| Temperature/K | 95(20) |
| a/Å | 9.681(3) |
| b/Å | 23.395(2) |
| c/Å | 23.285(8) |
| α/° | 90.0 |
| β/° | 90.38(4) |
| γ/° | 90.0 |
| Volume/Å$^3$ | 5274(2) |

**Figure S3.** X-ray diffraction pattern of **1•$C_{60}$** grown from toluene. (a) Image of crystal mounted on loop, (b) frame during data collection, and (c) table showing unit cell parameters. A full dataset could not be collected due to weak diffraction limitations.

a. b.

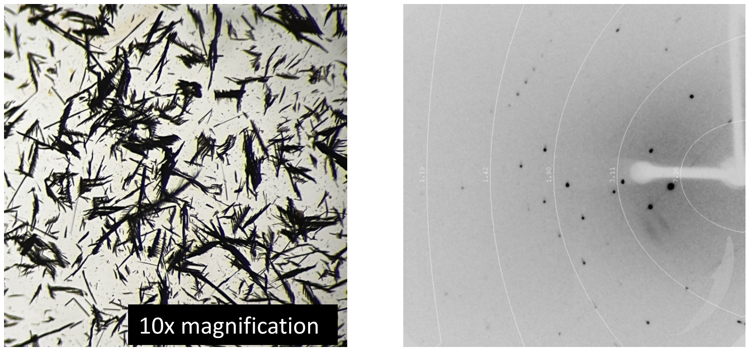


c.

| Crystal data and structure refinement for Co4S4 C60_auto. | |
|---|---|
| Identification code | **2•C$_{60}$** |
| Temperature/K | 104 |
| a/Å | 9.627(5) |
| b/Å | 23.223(13) |
| c/Å | 23.194(13) |
| α/° | 90.11(4) |
| β/° | 89.99(4) |
| γ/° | 90.07(4) |
| Volume/Å$^3$ | 5185(5) |

**Figure S4.** X-ray diffraction pattern of **2•C$_{60}$** grown from toluene. (a) Micrograph of crystals on a glass slide in paratone oil, (b) frame during data collection, and (c) table showing unit cell parameters. A full dataset could not be collected due to weak diffraction limitations.

## IV. Energy dispersive X-ray spectrograph of 1•C$_{60}$

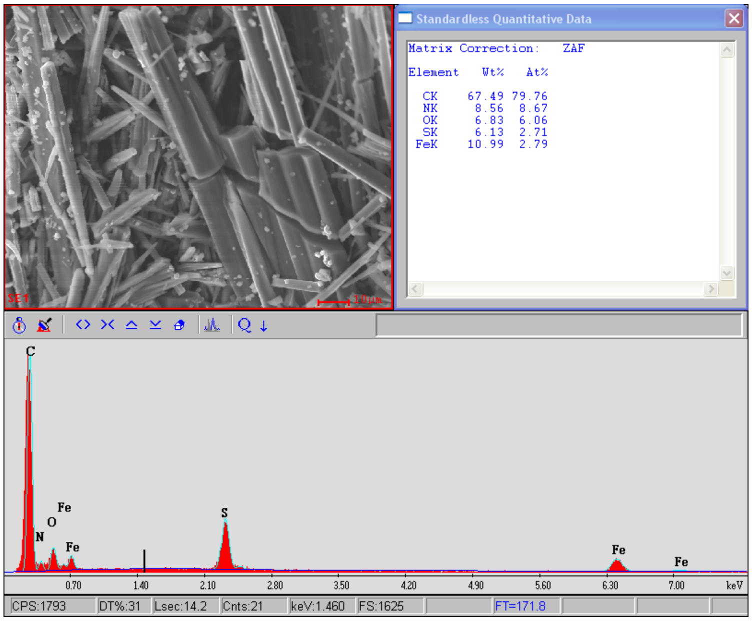


**Figure S5.** EDS survey scan and elemental composition of **1•C$_{60}$**. The spectrum was collected using carbon tape as a background.

### V. Photographs of Dissolved Superatomic Crystals 1•$C_{60}$

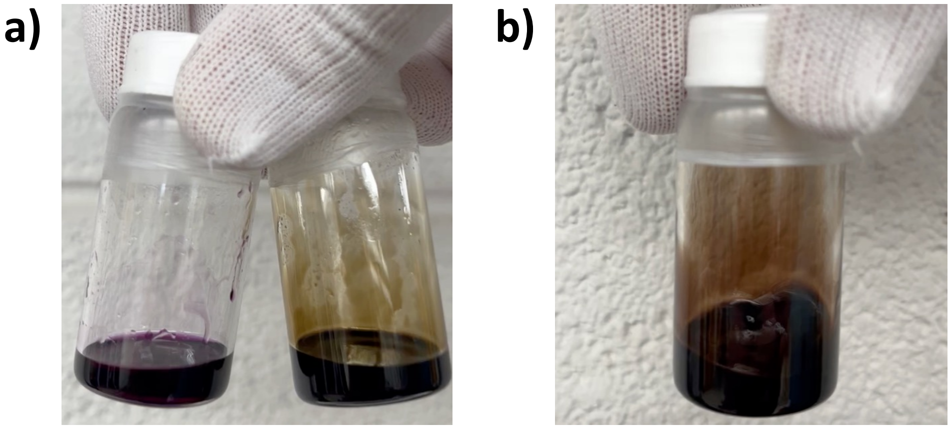


**Figure S6.** Images of $C_{60}$ and **1** dissolved in *o*-dichlorobenzene before mixing (a) and one hour after mixing (b).

### VI. Electrochemical analysis of 1•$C_{60}$ and 2•$C_{60}$

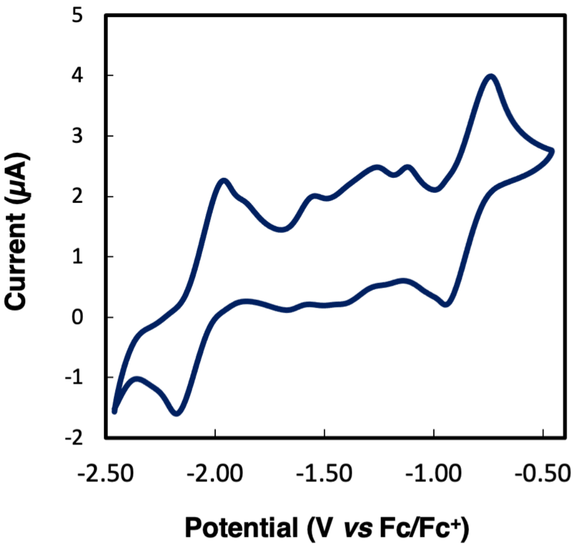


**Figure S7.** Cyclic voltammogram of **1** (5 mM) in THF measured with a glassy carbon working electrode, Pt counter electrode, and Pt-wire pseudo-reference electrode. Measured at 100 mV/s with $TBAPF_6$ as the supporting electrolyte.

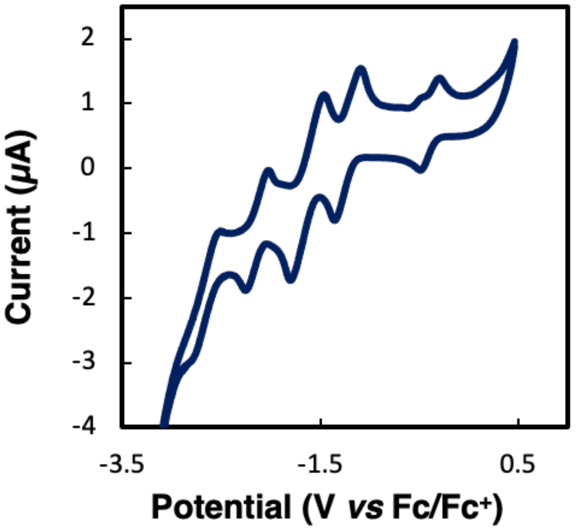


**Figure S8.** Cyclic voltammogram of **2•C$_{60}$** recorded at 25 mV s$^{-1}$ in THF with TBAPF$_6$ using a glassy carbon working electrode, Pt counter electrode, and Pt-wire pseudo-reference electrode.

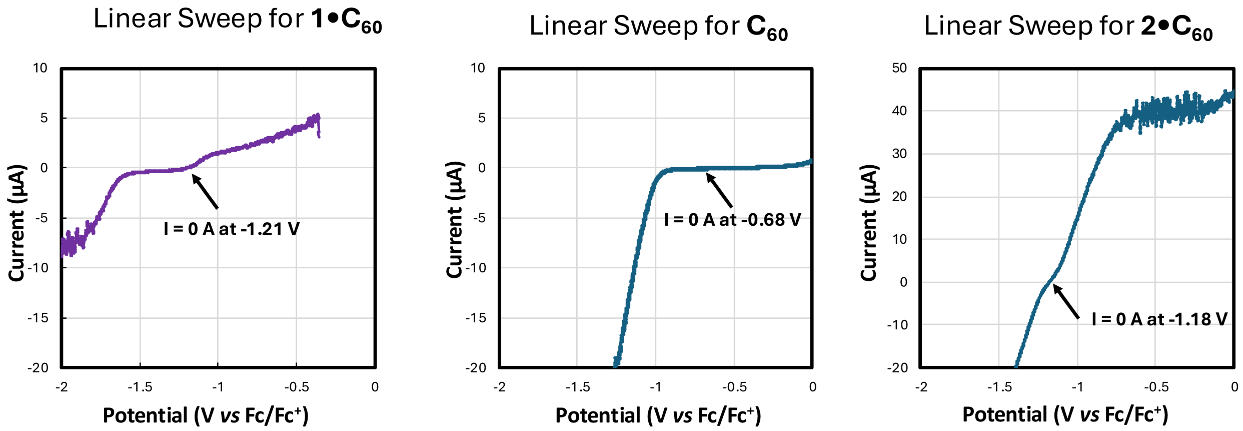


**Figure S9.** Linear sweep voltammograms of recorded at 20 mV s$^{-1}$ with TBAPF$_6$ using a glassy carbon working electrode, Pt counter electrode, and Pt-wire pseudo-reference electrode. **1•C$_{60}$** and **2•C$_{60}$** were recorded in THF. C$_{60}$ was recorded in toluene. The solutions were stirred during the measurement. The voltage values at zero current are listed on the plots.

## VII. UV-Vis-NIR Absorption Spectroscopy of 2•$C_{60}$

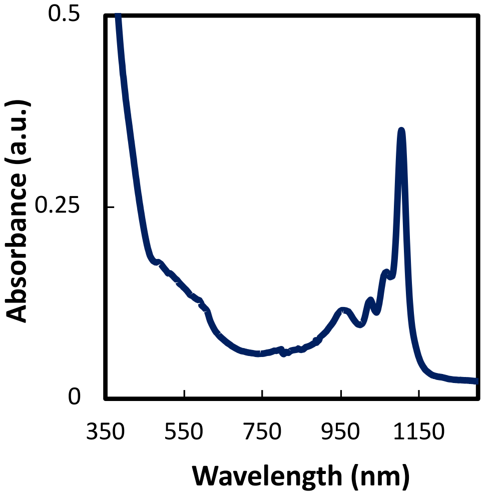


**Figure S10.** UV-Vis absorption spectrum of **2•$C_{60}$** in *o*-dichlorobenzene.

## VIII. NMR Spectroscopy of Dissolved Superatomic Crystals.

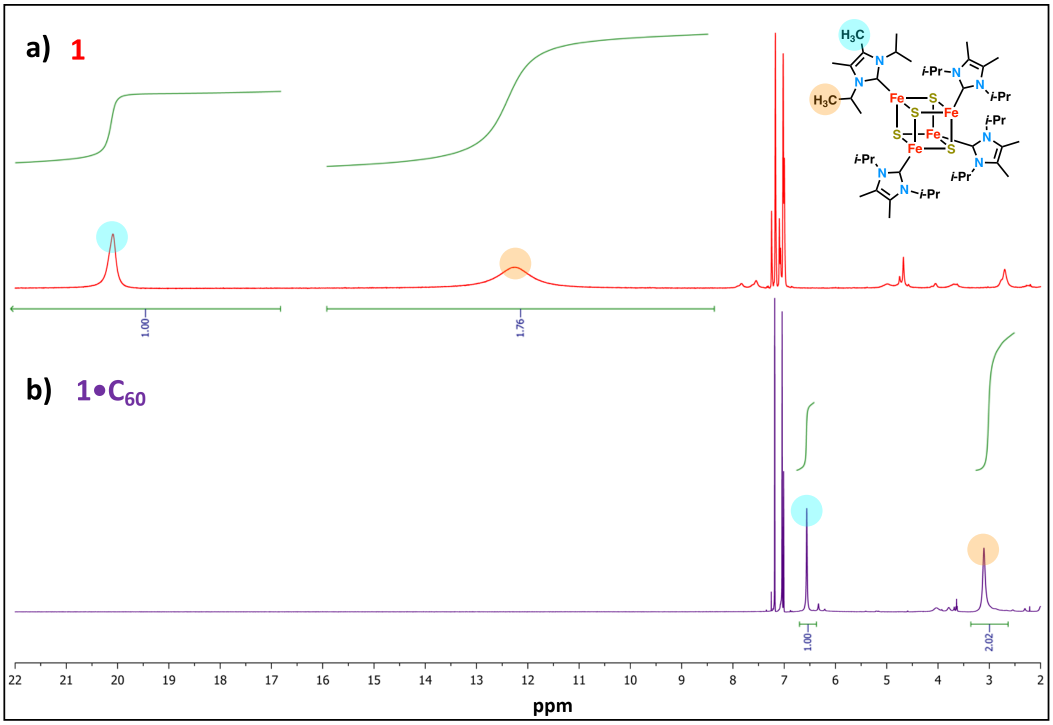


**Figure S11.** NMR spectroscopy studies of **1•$C_{60}$**. (a) $^1$H NMR spectra of **1** and (b) **1•$C_{60}$** in chlorobenzene-$d_5$. All spectra recorded at 500 MHz.

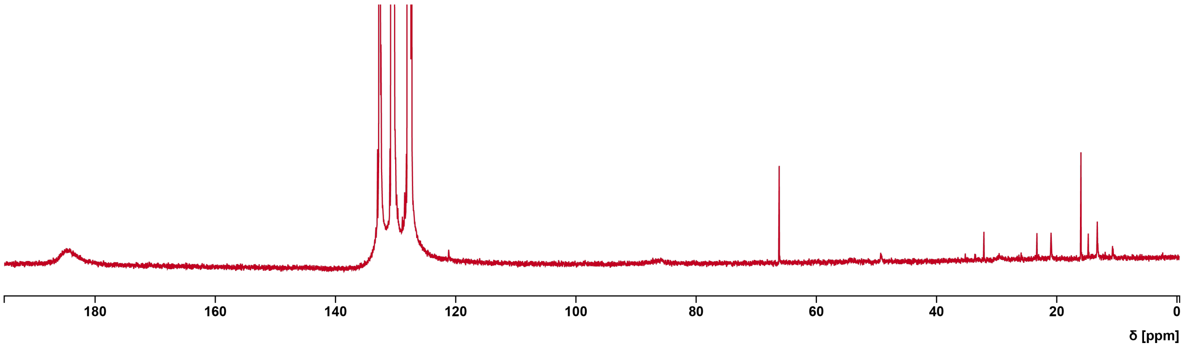


**Figure S12.** $^{13}$C NMR spectrum of **2•C$_{60}$** in chlorobenzene-$d_5$ recorded at 500 MHz.

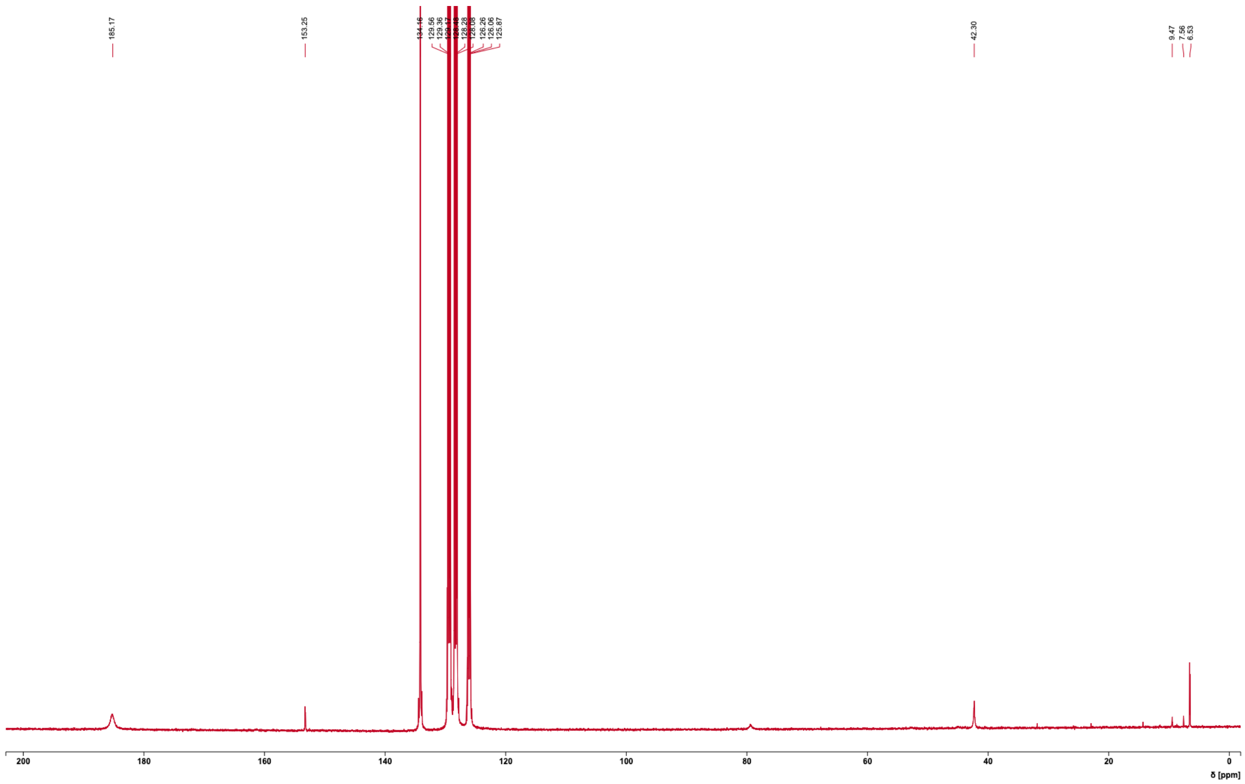


**Figure S13.** $^{13}$C NMR spectrum of **1•C$_{60}$** in chlorobenzene-$d_5$ recorded at 500 MHz.

## IX. Characterization of MCOP-2•$C_{60}$

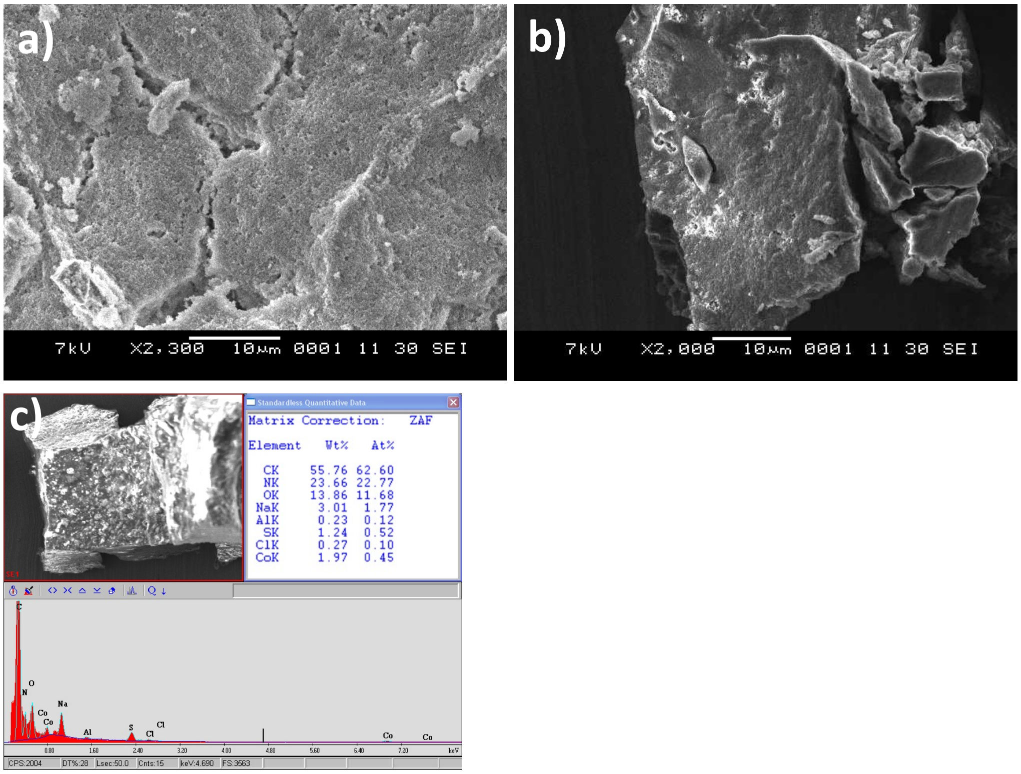


**Figure S14**. SEM micrographs of **MCOP-2** (a)**, MCOP-2•$C_{60}$** (b), and EDX of **MCOP-2•$C_{60}$** (c).

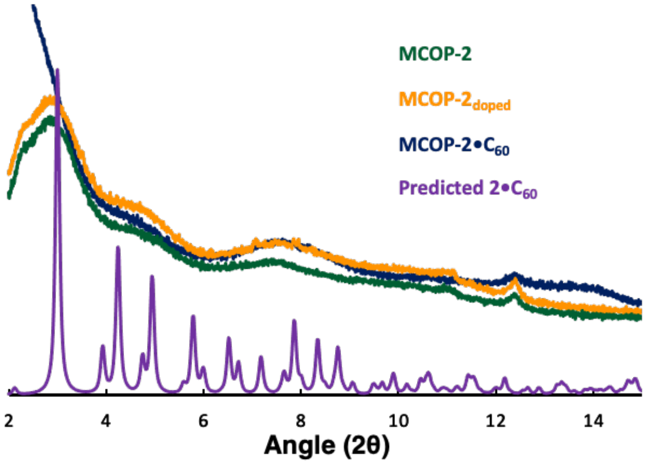


**Figure S15.** Overlay of measured synchrotron PXRD patterns of **MCOP-2**, **MCOP-2•$C_{60}$**, **MCOP-2$_{doped}$**, and simulated pattern of **2•$C_{60}$**. The data was collected on a beamline using an average wavelength (λ) of 0.6086 Å.

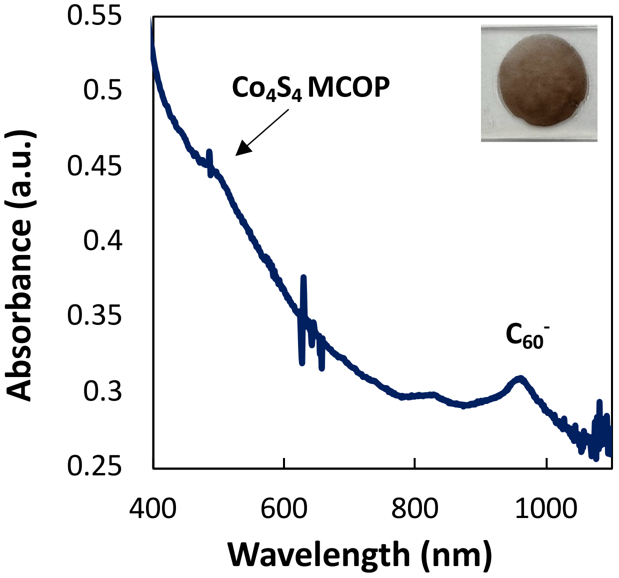


**Figure S16**. UV-Vis absorption spectrum of **MCOP-2•$C_{60}$** (inset image **MCOP-2•$C_{60}$** in epoxy).

## X. Characterization of MCOP-1

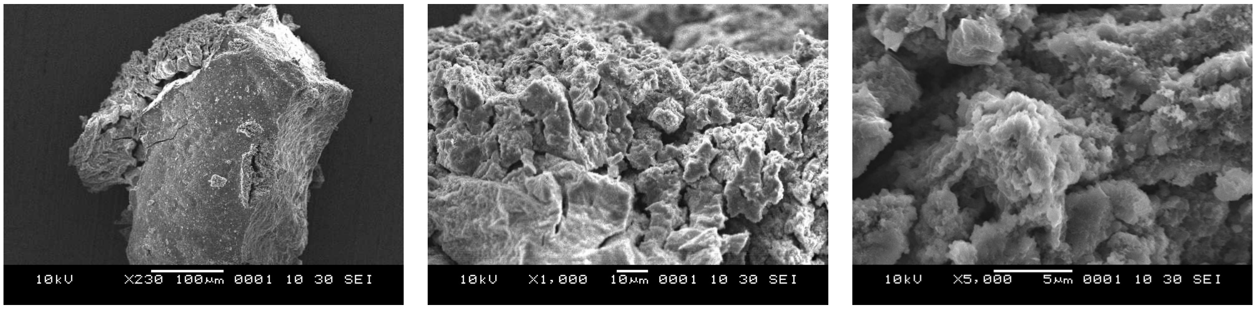


**Figure S17**. SEM micrographs of **MCOP-1**.

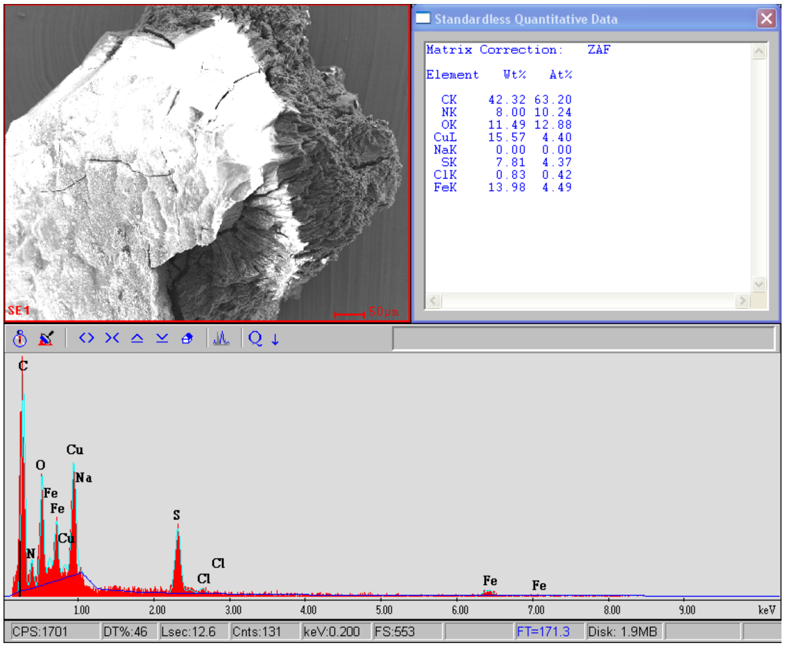


**Figure S18.** EDS survey scan and elemental composition of **MCOP-1** collected using copper tape as a background.

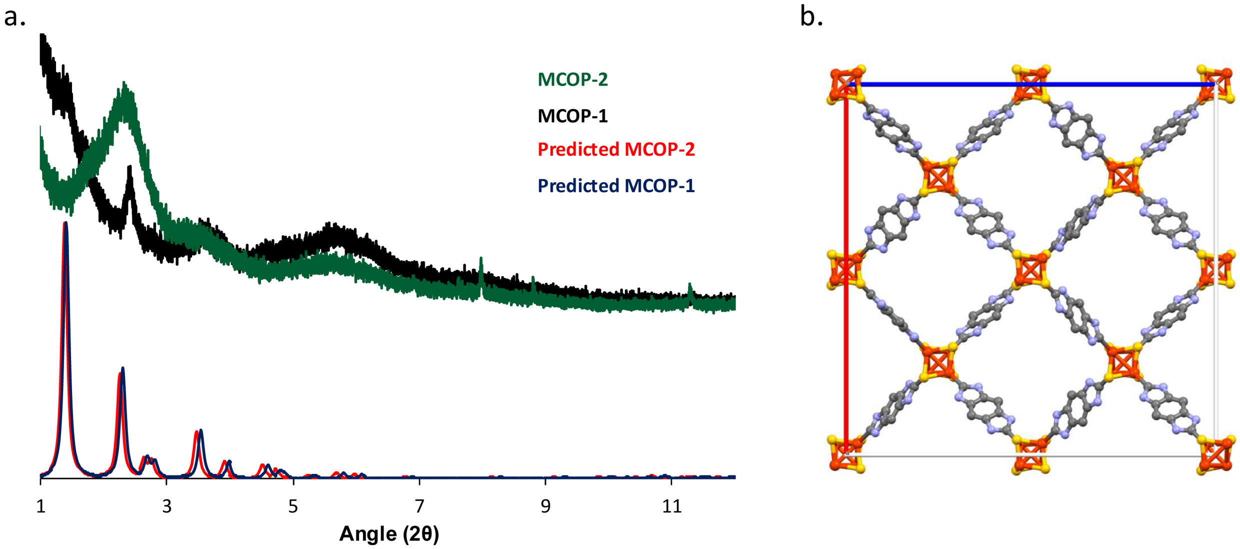


**Figure S19.** (a.) Synchrotron powder X-ray diffraction scans of **MCOP-1** (black) and **MCOP-2** (green) collected with λ = 0.4581 Å and simulated patterns of **MCOP-1** and **MCOP-2**; (b.) model of **MCOP-1** used for predicted pattern. Ethyl groups and hydrogen atoms omitted for clarity.

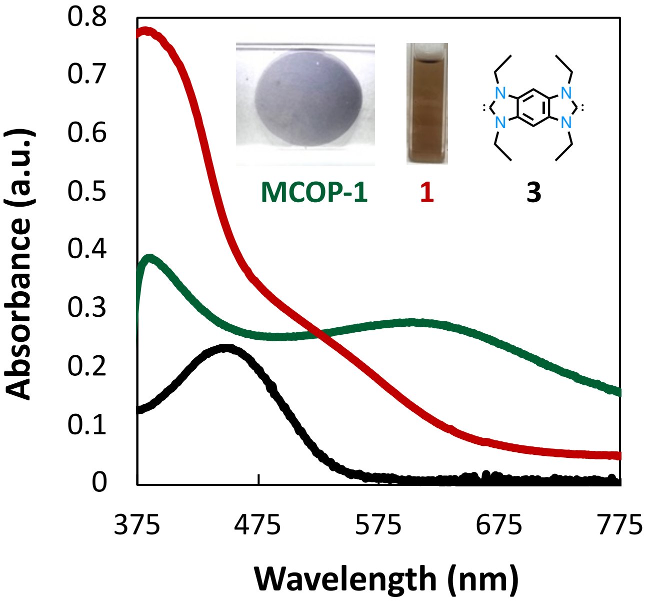


**Figure S20.** Normalized UV-Vis absorption spectra of **1, MCOP-1,** and **3** (inset images of a solution of **1** in THF and **MCOP-1** in epoxy).

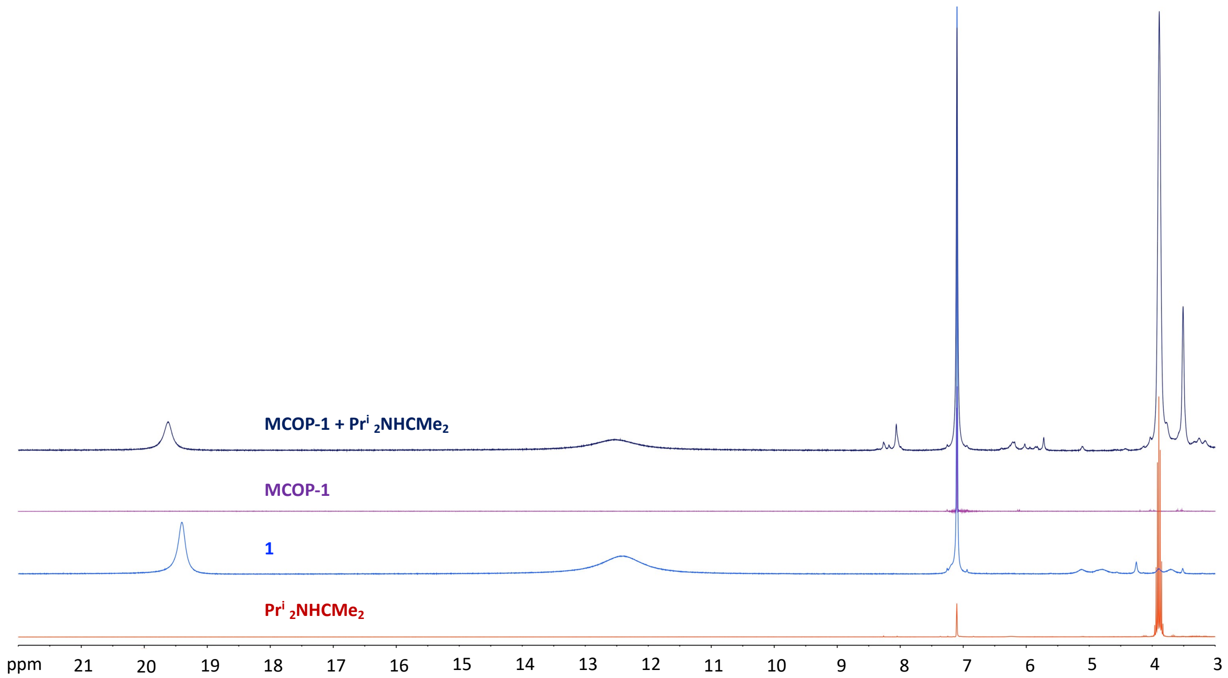


**Figure S21**. Depolymerization of **MCOP-1:** NMR spectra overlay of **MCOP-1** (purple); cluster **1** (light blue); ligand $Pr^i{}_2NHCMe_2$ (red); and mixture of **MCOP-1** and $Pr^i{}_2NHCMe_2$ after depolymerization (dark blue) in $C_6D_6$. The peak at 3.4 ppm and other small impurities present in the spectrum after depolymerization likely arise from the formation of various isomers of polyenetetraamine that are in equilibrium with benzo-bis-imidazolylidene **3**.[4]

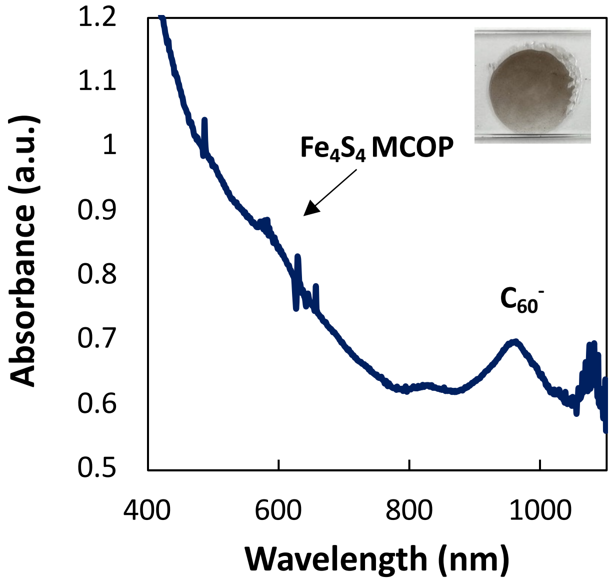


**Figure S22**. UV-Vis absorption spectrum of **MCOP-1•C$_{60}$** (inset image **MCOP-1•C$_{60}$** in epoxy).

**XI. UV-Vis-NIR studies of MCOPs Postsynthetically Doped with Fullerene.**

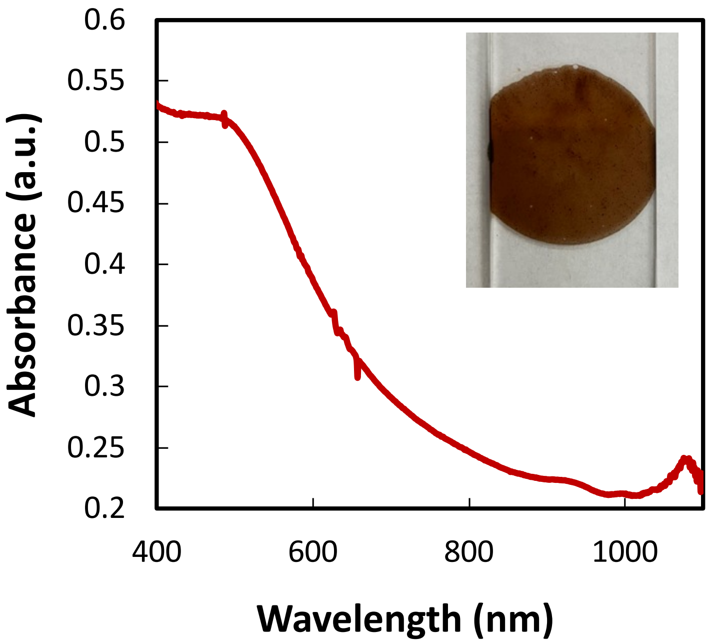


**Figure S23**. UV-Vis-NIR spectrum of **MCOP-2** after soaking in a saturated solution of C$_{60}$ for 24 hours. The spectrum was collected on the polymer, which is sealed in epoxy between two glass slides to protect the sample from air exposure. Inset shows an image of the sample.

## XII. Images of MCOP Postsynthetically Doped with Fullerene.

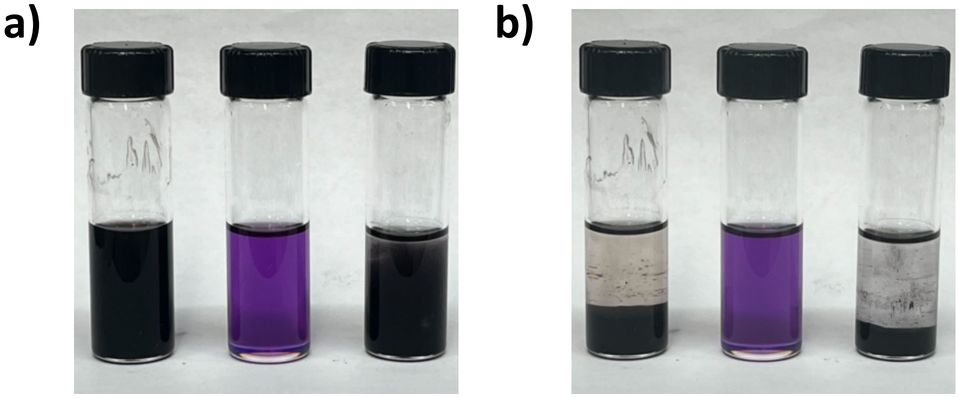


**Figure S24**. (a) Initial photographs of **MCOP-2** suspended in a solution of toluene with $C_{60}$ (left), $C_{60}$ in toluene (middle), and **MCOP-2** in toluene (right). (b) Photographs of the same solutions 24 hours later. Images shown are meant to illustrate color change upon reaction/adsorption of $C_{60}$. MCOP samples used for conductivity measurements were doped with excess fullerene to ensure maximum saturation.

## XII. Electrical Conductivity Measurements

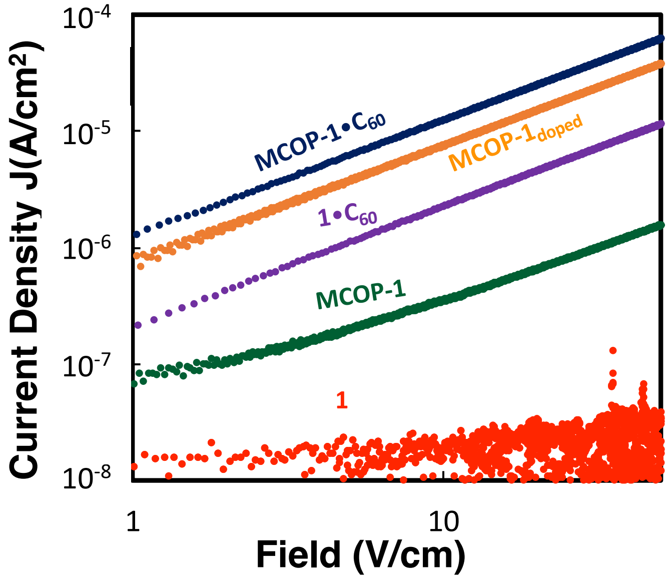


**Figure S25**. Electrical properties of pressed pellets of Fe-based materials. Plots of current density versus electric field strength (*J*–*E*) curves at 297 K.

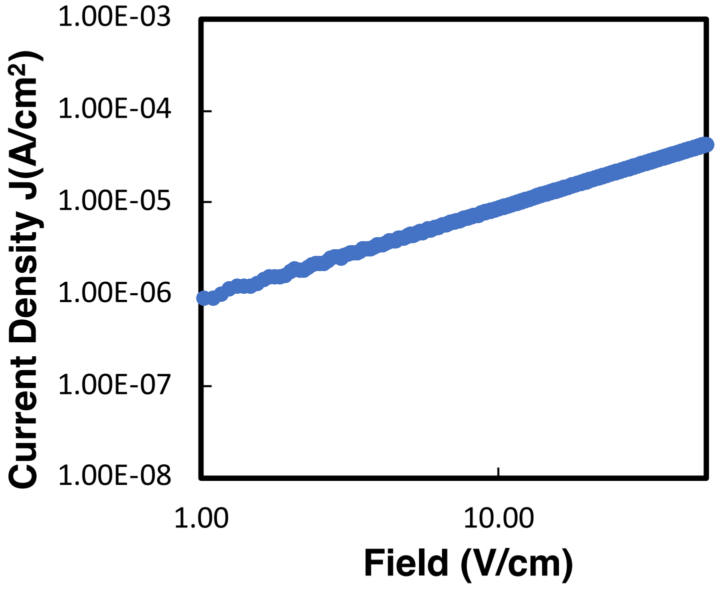


**Figure S26**. Electrical properties of pressed pellets of **MCOP-2•BF₄**. Plots of current density versus electric field strength (*J*–*E*) curves at 297 K.

**Table S1 Conductivity values of related reference materials**

| Material | Conductivity (mS/cm) |
|---|---|
| $C_{60}$[7]<br>$C_{60}$ (film)[8, 9] | $10^{-11}$<br>$10^{-5}$ |
| Cobalt Sulfide[10] | $CoS_2$ (cattierite) = 5 x $10^6$ |
| Iron Sulfide[10] | Pyrite = 5.75 x $10^2$<br>Marcasite $FeS_2$ = $10^4$<br>Pyrrhotite $Fe_7S_8$ = 100 to $10^7$<br>Troilite FeS = 100 to $10^7$ |
| Cobalt Metal | $1.7 \times 10^8$ |
| Iron Metal | $10^7$ |
| Cobalt Hydroxide[11] | $8.6 \times 10^{-5}$ |

## XIV. X-ray Diffraction Procedures and Tables

X-ray diffraction data were collected using an Agilent/Oxford Diffraction Gemini A Ultra diffractometer. The diffractometer was equipped with a sealed-tube Mo source with a graphite monochromator (λ=0.71073 Å) and a sealed-tube Cu source with graphite focusing optics (λ=1.54184 Å). In all cases data were collected to better than 0.8 Å resolution.

The CrysAlisPro diffractometer software[12] provided intensity data corrected for Lorentz, polarization, and absorption effects. Preliminary structure solutions were found with the AutoChem software package. Final structures were solved with SHELXT[13] (direct methods) and refined with SHELXL[14], using the Olex2 software package[15]. Anisotropic refinement for all non-hydrogen atoms was performed except where noted. Hydrogen atoms were placed in calculated positions.

The crystal structure refinement for **2•$C_{60}$** was complex because the fullerene is disordered over a fourfold symmetry element. Refinement details are given in the cif file (_refine_special_details). Briefly, the geometry for the disordered $C_{60}$ fragment was introduced from a reference structure and the orientation was identified by a Monte Carlo search. Subsequently the geometry was relaxed with the aid of DFIX restraints on all 1,2- and 1,3-distances.

Publication tables and graphics were generated with the WinGX[16] suite of programs.

**Table S2 Crystal data and structure refinement for 2•$C_{60}$.**

| Compound | 2·$C_{60}$ |
|---|---|
| Formula | $C_{128}H_{100}Cl_4Co_4N_8S_4$ |
| MW | 2255.91 |
| Space group | *P*-4*n*2 |
| ***a*** (Å) | 23.2168(7) |
| ***b*** (Å) | 23.2168(7) |
| ***c*** (Å) | 9.5988(4) |
| ***α*** (°) | 90 |
| ***β*** (°) | 90 |
| ***γ*** (°) | 90 |
| V (Å$^3$) | 5173.9(3) |
| Z | 2 |
| $\rho_{calc}$ (g cm$^{-3}$) | 1.448 |
| λ (Å) | 0.71073 |
| T (K) | 108 |
| $d_{min}$ (Å) | 0.72 |
| μ(mm$^{-1}$) | 0.872 |
| Data | 6442 |
| Restraints | 415 |
| Parameters | 510 |
| $R_1$(obs) (%) | 5.09 |
| $R_1$(all) (%) | 6.29 |
| S | 1.065 |
| Peak, hole (e$^-$ Å$^{-3}$) | .52, -.38 |

**Table S3. Bond Length Comparison $Co_4S_4$ Structures in Different Oxidation States**

| $Co_4S_4(NHC)_4$ | | $Co_4S_4(NHC)_4^{+1}$ | | 2•$C_{60}$ | |
|---|---|---|---|---|---|
| ***Co-Co*** | ***Co-S*** | ***Co-Co*** | ***Co-S*** | ***Co-Co*** | ***Co-S*** |
| 2.7227(7) | 2.2446(7) | 2.6606(6) | 2.2307(9) | 2.6506(12) | 2.2174(14) |
| 2.6782(6) | 2.2558(8) | 2.6221(6) | 2.2206(9) | 2.6603(12) | 2.2110(14) |
| 2.6792(5) | 2.2623(7) | 2.6631(6) | 2.2035(9) | 2.6603(12) | 2.2305(14) |
| 2.6771(6) | 2.2542(7) | 2.6840(6) | 2.2092(9) | | |
| 2.7106(6) | 2.2401(8) | 2.6822(6) | 2.2271(9) | | |
| 2.6792(5) | 2.2463(8) | 2.6575(6) | 2.2317(9) | | |
| | 2.2739(8) | | 2.2268(9) | | |
| | 2.2599(8) | | 2.2087(9) | | |
| | 2.2447(8) | | 2.2041(9) | | |
| | 2.2527(8) | | 2.2095(9) | | |
| | 2.2644(7) | | 2.2230(9) | | |
| | 2.2502(8) | | 2.2378(9) | | |
| | | | | | |
| ***AVG*** | ***AVG*** | ***AVG*** | ***AVG*** | ***AVG*** | ***AVG*** |
| **2.6913** | **2.2541** | **2.6615** | **2.2193** | **2.6566** | **2.2196** |

## XV. References


(1) Booth, G.; Chatt, J. 402. The reactions of carbon monoxide and nitric oxide with tertiary phosphine complexes of iron (II), cobalt (II), and nickel (II). *Journal of the Chemical Society (Resumed)* **1962**, 2099-2106.

(2) Deng, L.; Holm, R. H. Stabilization of fully reduced iron-sulfur clusters by carbene ligation: the [FenSn]0 oxidation levels (n = 4, 8). *J Am Chem Soc* **2008**, *130* (30), 9878-9886. DOI: 10.1021/ja802111w From NLM Medline.

(3) Deng, L.; Bill, E.; Wieghardt, K.; Holm, R. Cubane-type Co4S4 clusters: synthesis, redox series, and magnetic ground states. *J Am Chem Soc* **2009**, *131* (31), 11213-11221.

(4) Kamplain, J. W.; Bielawski, C. W. Dynamic covalent polymers based upon carbene dimerization. *Chemical communications* **2006**, (16), 1727-1729.

(5) Gillen, J. H.; Moore, C. A.; Vuong, M.; Shajahan, J.; Anstey, M. R.; Alston, J. R.; Bejger, C. M. Synthesis and disassembly of an organometallic polymer comprising redox-active Co 4 S 4 clusters and Janus biscarbene linkers. *Chemical Communications* **2022**, *58* (31), 4885-4888.

(6) Wudl, F.; Bryce, M. R. Apparatus for two-probe conductivity measurements on compressed powders. *Journal of Chemical Education* **1990**, *67* (8), 717.

(7) Ivetić, M.; Mojović, Z.; Matija, L. Electrical conductivity of fullerene derivatives. In *Materials Science Forum*, 2003; Trans Tech Publications, Switzerland: Vol. 413, pp 49-52.

(8) Hamed, A.; Sun, Y.; Tao, Y.; Meng, R.; Hor, P. Effects of oxygen and illumination on the in situ conductivity of C 60 thin films. *Physical Review B* **1993**, *47* (16), 10873.

(9) Haddon, R.; Hebard, A.; Rosseinsky, M.; Murphy, D.; Duclos, S.; Lyons, K.; Miller, B.; Rosamilia, J.; Fleming, R.; Kortan, A. Conducting films of C60 and C70 by alkali-metal doping. *Nature* **1991**, *350* (6316), 320-322.
(10) Pearce, C. I.; Pattrick, R. A.; Vaughan, D. J. Electrical and magnetic properties of sulfides. *Reviews in Mineralogy and Geochemistry* **2006**, *61* (1), 127-180.
(11) Shakir, I.; Almutairi, Z.; Shar, S. S.; Nafady, A. Synthesis of Co (OH) 2/CNTs nanocomposite with superior rate capability and cyclic stability for energy storage applications. *Materials Research Express* **2020**, *7* (12), 125501.
(12) Rigaku, O. CrysAlisPro software system. *Version* **2018**, *1* (38.41), l.
(13) Sheldrick, G. M. Crystal structure refinement with SHELXL. *Crystal Structure Communications* **2015**, *71* (1), 3-8.
(14) Sheldrick, G. M. A short history of SHELX. *Foundations of crystallography* **2008**, *64* (1), 112-122.
(15) Dolomanov, O. V.; Bourhis, L. J.; Gildea, R. J.; Howard, J. A.; Puschmann, H. OLEX2: a complete structure solution, refinement and analysis program. *Applied Crystallography* **2009**, *42* (2), 339-341.
(16) Farrugia, L. J. WinGX and ORTEP for Windows: an update. *Applied Crystallography* **2012**, *45* (4), 849-854.
